# A Network Architecture for Bidirectional Neurovascular Coupling in Rat Whisker Barrel Cortex

**DOI:** 10.3389/fncom.2021.638700

**Published:** 2021-06-15

**Authors:** Bhadra S. Kumar, Aditi Khot, V. Srinivasa Chakravarthy, S. Pushpavanam

**Affiliations:** ^1^Computational Neuroscience Laboratory, Department of Biotechnology, Bhupat and Jyoti Mehta School of Biosciences, Indian Institute of Technology Madras, Chennai, India; ^2^Department of Chemical Engineering, Purdue University, West Lafayette, IN, United States; ^3^Department of Chemical Engineering, Indian Institute of Technology Madras, Chennai, India

**Keywords:** bidirectional network model, hypoxia-ischemia, neurovascular coupling, plasticity, whisker barrel cortex

## Abstract

Neurovascular coupling is typically considered as a master-slave relationship between the neurons and the cerebral vessels: the neurons demand energy which the vessels supply in the form of glucose and oxygen. In the recent past, both theoretical and experimental studies have suggested that the neurovascular coupling is a bidirectional system, a loop that includes a feedback signal from the vessels influencing neural firing and plasticity. An integrated model of bidirectionally connected neural network and the vascular network is hence required to understand the relationship between the informational and metabolic aspects of neural dynamics. In this study, we present a computational model of the bidirectional neurovascular system in the whisker barrel cortex and study the effect of such coupling on neural activity and plasticity as manifest in the whisker barrel map formation. In this model, a biologically plausible self-organizing network model of rate coded, dynamic neurons is nourished by a network of vessels modeled using the biophysical properties of blood vessels. The neural layer which is designed to simulate the whisker barrel cortex of rat transmits vasodilatory signals to the vessels. The feedback from the vessels is in the form of available oxygen for oxidative metabolism whose end result is the adenosine triphosphate (ATP) necessary to fuel neural firing. The model captures the effect of the feedback from the vascular network on the neuronal map formation in the whisker barrel model under normal and pathological (Hypoxia and Hypoxia-Ischemia) conditions.

## Introduction

The brain is one of the most energy-intensive organs in the human body, consuming around 20% of the total cardiac output even though it constitutes just 2% of the total body weight (Sokoloff et al., 1955; Clark and Sokoloff, 1999; Raichle and Gusnard, 2002). Despite the high energy demands of this organ, there seems to be no significant energy reservoir in the neural tissue and energy is provided in an “on demand” fashion by an increase in blood flow in proximal blood vessels (functional hyperemia) (Raichle and Shepherd, 2014) as a response to neural activity. Classical accounts of neurovascular coupling describe the interaction between neurons and cerebral micro-vessels as a unidirectional influence from neurons to vessels (Buxton and Frank, 1997; Buxton et al., 2004). However, the dependence of the neurons on the energy substrates delivered by the vessels raises the possibility of a reverse signal emanating from the vessels and influencing neural dynamics.

The possibility of a retrograde signal from the vessels to the neurons was first discussed under the name of the hemoneural hypothesis by Moore and Cao (2008). Sirotin and Das (2009) showed that under certain conditions the vascular response precedes the neural response. Leithner and Royl (2014) demonstrated a decorrelation between blood volume changes and neural oxygen demand. Such studies strengthen the case for a revision of the notion of a master-slave relationship between neurons and cerebral vessels. The study by Filosa and colleagues (Filosa et al., 2006; Kim et al., 2016) suggested that vessels can have a direct influence on the neurons not necessarily by releasing energy substrates, but by other mechanisms like, for example, the mechanical pressure exerted by the vessels transduced into electrical signals in the neurons.

Even though the explicit vascular signal feedback to neurons is debated, it is well understood that the metabolic feedback from the cerebral vessels is crucial in sustaining neural activity (Laughlin, 2001; Niven and Laughlin, 2008; Hasenstaub et al., 2010; Yu and Yu, 2017; Vergara et al., 2019). This motivated an intriguing question: “What are the vascular influences, if any, on neural performance?” The possibility of a retrograde influence of the cerebral vessels on the neurons was also explored from a computational modeling perspective. Gandrakota et al. (2010) presented a model of the neuro-glio-vascular network as a bidirectional associative memory with information flowing both in neurovascular and vasculoneural directions. A detailed biophysical model of a single neuron-astrocyte- vessel system by Chander and Chakravarthy (2012) showed how the slow vasomotor rhythms can modulate neural firing, thereby reinforcing the idea of a retrograde signal. This model was later simplified and extended to a network level in Chhabria and Chakravarthy (2016), where the vascular and glial structures were clubbed into a single gliovascular unit whose output determines the energy level of neurons. In a neurovascular network model proposed by Philips et al. (2016), desynchronized vascular rhythms were found to improve the efficiency of learning in a neural network trained like an autoencoder. Among the aforementioned set of models, there are either detailed biophysical models at the single unit level or abstract models at a network level. There is a need to create detailed neurovascular models, incorporating key biophysical elements and exploring, preferably at a network level, the consequences of a retrograde signal from the vessels to the neurons on neuronal activity and plasticity. This last objective forms the motivation of the present study. In this paper, we develop a network level architecture for neurovascular interaction where the activity of neurons not only influences the vascular activity in the forward direction but also depends on the feedback from the proximal vessels based on their volume and oxygen saturation. We explore how to establish a bidirectional coupling between a network of laterally connected rate coded neuronal units and a network of vascular units inspired from the vascular anatomical network (VAN) model (Boas et al., 2008). On establishing a bidirectional neurovascular coupling, the characteristics of vascular network like inlet oxygen saturation and diameter of the Pial artery are altered to simulate conditions of hypoxia and ischemia. Since the neural network receives continuous feedback from vascular network, any training or retraining which happens in the neural network in this model is influenced by the vascular feedback. This enables the model to bring out the possible variations in the characteristics of the neural network such as neural plasticity under altered vascular feedback. It is almost impossible to carry out such an exploration using detailed biophysical neurovascular network. Since there is a growing awareness in the recent years that neurodegenerative diseases owe their origin to disrupted neurovascular coupling (Iadecola, 2017; Muddapu et al., 2020), there is a great need to understand the influence of vascular feedback at a network level.

Neurovascular coupling is believed to be made possible through direct neuron-vessel communication (Tong and Hamel, 2000) and also through a pathway involving astrocytes (Attwell et al., 2010; Mishra et al., 2016). Astrocytes are also believed to play a role in providing metabolic feedback to the neurons along with direct metabolic feedback from the vessels (Magistretti, 2011; Magistretti and Allaman, 2018). In order to simplify the various neurovascular coupling pathways, we assume a unified variable to represent all the vasodilatory signals that reach the vessel *via* various pathways. Similarly, the entire metabolic feedback received by the neurons *via* various pathways is represented by one single variable which largely depends on oxidative phosphorylation (Hall et al., 2012; Yellen, 2018) to yield energy substrates.

**FIGURE 1:**
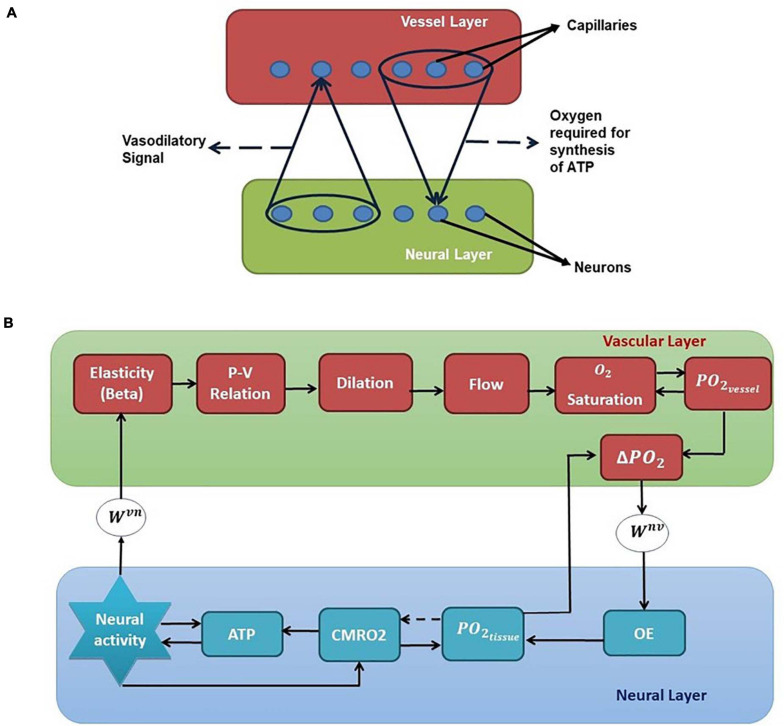
**(A)** Bidirectional connection between neural and vascular layers. Neurons send afferent vasodilatory signals to vessels and vessels send oxygen back to the neural layer for oxidative phosphorylation. **(B)** A schematic of the interactions among various quantities involved in neurovascular coupling. Beta (compliance factor of the vessel, β). P, pressure; V, volume; S, saturation of oxygen in the blood; PO_2_, partial pressure of oxygen; OE, oxygen extraction; CMRO_2_, cerebral metabolic rate of oxygen; *W*^*vn*^ and *W*^*nv*^, Gaussian weights; ATP, adenosine triphosphate.

We have selected the rat whisker barrel cortex as the model system for which we develop a neurovascular network model. In order to simulate the vascular influence on neural activity and plasticity, a network level model of neural layer and a network of vascular structure that perfuses this neural layer at the level of capillaries are required. The vascular network which perfuses the whisker barrel cortex layer L4 is modeled using a three-dimensional vascular tree architecture that branches and penetrates the neural layer at the level of capillaries. The anatomy of the vascular network is inspired from the studies of Tsai et al. (2009) and Blinder et al. (2013) and the vascular dynamics are similar to that of the VAN model of Boas et al. (2008). The pressure-volume relationship in the blood vessel is assumed to be linear with the linearity coefficient modifiable by neural activity. The activity of a neuron influences the vessels in its neighborhood, while the neuron, in turn, is nourished by the vessels in a neighborhood thereby making the neurovascular network bidirectional (Figure 1A).

The model allows control of arteriolar dilation and capillary dilation independently which would help to incorporate the studies that show that while the arteriolar dilation is mediated by direct neural influence, the capillary dilation is mostly mediated by astrocytes (Biesecker et al., 2016; Mishra et al., 2016). The concept of arteriolar dilation, rather than venous dilation, being the primary response to short duration neural stimulation has been explored before in modeling studies (Kim et al., 2013; Kim and Ress, 2016). Since the pathways involved in capillary dilation (Biesecker et al., 2016; Mishra et al., 2016) differ from those involved in arteriolar dilation (Nizar et al., 2013; Bonder and McCarthy, 2014), it is important that the model provides provision to control them independently.

The major question concerning neurovascular coupling is: what does the neural activity alter at the vessels, or how does the neurovascular coupling manifest? A few neurovascular coupling models incorporate the neural activity in the function that defines the cerebral blood flow, thereby regulating the cerebral blood flow directly (Drysdale et al., 2010; Kim et al., 2013; Kim and Ress, 2016). A few others directly change the resistance of the vascular unit to reflect vasodilation (Boas et al., 2008). But we believe that neural activation can be thought to alter pressure—volume relationship of the vessel. This is in line with models of neural activation of even the skeletal muscle. According to the classic Hill’s model of the skeletal muscle (Hill, 1938), the main difference between a passive and an active (activated by neural inputs) muscle is an altered tension-length relationship. (Under non-steady state conditions, the relation between Tension-length-velocity is described.) In an active muscle, a greater tension is created at the same muscle length. Tension-length relationship is basically a manifestation of elasticity and therefore any change in that relationship can be treated as a change in effective elasticity. Furthermore, since muscle is viscoelastic, in its lumped models, it is modeled using a combination of spring(s) and a damper. The Hill’s modeling approach was also extended to models of smooth muscle (Warshaw and Fay, 1983; Warshaw, 1987). The same principles are applied in modified form even when modeling the vascular smooth muscle. In this case, one does not talk about length-tension relationship but about the pressure-volume relationship. In the current model, the vessel activation changes the relationship between volume and pressure, which is quantified in terms of compliance factor instead of elasticity.

In most of the vascular models, the pressure-volume relationship is considered non-linear (Mandeville et al., 1999; Drysdale et al., 2010). But for simplicity, we assume that the pressure and volume follow a linear relationship defined by a proportionality constant, (β), which we term as compliance factor in this model. We incorporate the influence of the neural activity in the vessel such that it causes a transient change in this compliance factor (β) of the vessel. This disturbs the pressure-volume equilibrium and results in vasodilation and increased cerebral blood flow. Once the neural activity subsides, the compliance factor (β) returns to the resting state value.

Hemodynamic response to neural activity is manifested in a number of different ways: as an increase in the local cerebral blood flow (rCBF), local cerebral blood volume (rCBV), and also as significant change in the oxygen saturation and concentration of glucose along with an increase of the metabolic rate of oxygen and glucose in the local neural tissues. All these changes reflect the neurovascular coupling between the neurons and blood vessels in that area. With the proposed model, the changes in cerebral blood flow, cerebral blood volume, saturation of oxygen and cerebral metabolic rate of oxygen can be observed. We explicitly plot only the hemoglobin concentration since we optimize the model to match the hemodynamic response observed in rat whisker barrel cortex in the experimental studies by Devor et al. (2005), where they observed the hemodynamic response by studying the change in concentration of total hemoglobin and oxygenated/deoxygenated hemoglobin.

Neural dynamics in the model is captured by the Laterally Interconnected Synergistically Self-Organizing Map (LISSOM) model which is a simple yet biologically plausible model to simulate cortical neural activity (Miikkulainen et al., 2005). The LISSOM model basically does feature extraction similar to the principal component analysis, thereby capable of forming topographic maps. In the current study, we train the LISSOM network to simulate the maps of the rat whisker barrel cortex. The parameters of the model are optimized to simulate experiments on neurovascular coupling under normal (Devor et al., 2005) and pathological (Ranasinghe et al., 2015) conditions. Spatial and temporal characteristics of the hemodynamic response in terms of the volume and oxygen saturation agree with experimental observations. The model is also able to capture the non-linearity of hemodynamic response. To explore the possibility of studying the vascular influence on neural layer properties, retraining of the neural layer is carried out under various conditions of vascular pathology. The model results are in agreement with experimental observations (Ranasinghe et al., 2015).

## Materials and Methods

**FIGURE 2:**
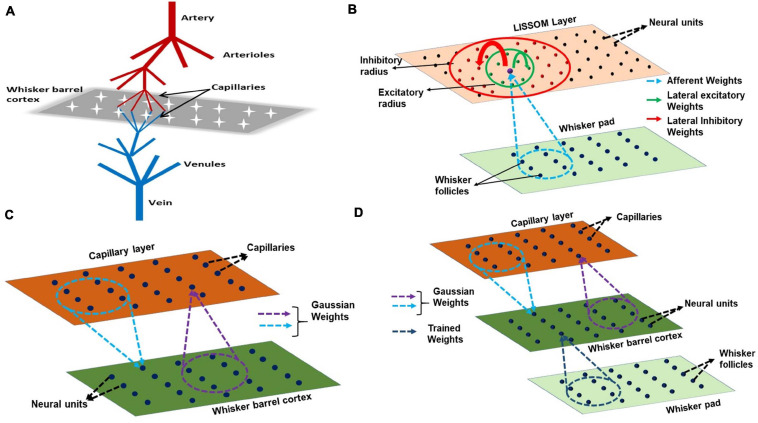
**(A)** A schematic of the model. The big pial artery branches to arterioles and they again branch to capillaries to perfuse the neurons. The neurons and vessels interact at the level of these capillaries. **(B)** LISSOM architecture. The neural unit receives weighted input from an area in the whisker pad (blue dots). It receives excitatory input from the immediate neighboring neural units (green dots) and inhibitory input from long range neural units (red dots). **(C)** The neurovascular connectivity. Each vessel receives vasodilatory information from an area of neural units (violet dotted circle). Each neural unit receives oxygen from a group of proximal vessels (blue dotted circle). **(D)** A schematic for complete interactions among the three layers, Input layer (whisker pad), Neural layer (whisker barrel cortex), and blood vessels (capillaries). The only trained weights are from input layer to whisker barrel cortex (dark blue dotted circle).

The proposed model for bidirectional neurovascular interaction consists of a three- dimensional vascular network which branches from a large pial artery into smaller arterioles and capillaries to perfuse the two-dimensional neural sheet at the level of capillaries (Figure 2A). The whisker stimulations act as input stimuli to the neural layer and its response is conveyed to the vascular layer in the form of vasoactive signals causing vessel dilation/contraction. The vascular response determines the release of “energy” to the neural layer, which controls the threshold of neural activation.

### Modeling the Input Layer

**FIGURE 3:**
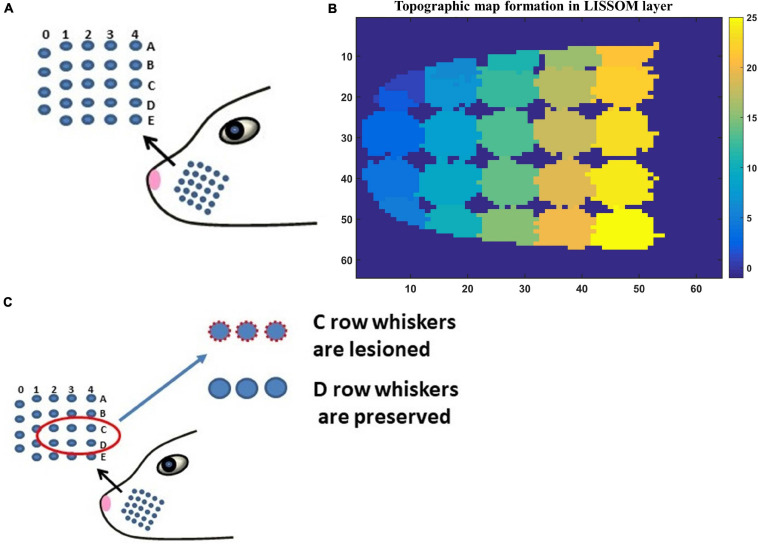
**(A)** The whisker pad of the rat. Each whisker is addressed using the row (A–E) and the column (0–4). **(B)** The topographic map formed in whisker barrel cortex. The whiskers are given numbers 1–24 such that, column 0 has whiskers 1–4; column 1 (5–9); column 2 (10–14); column 3 (15–19); column 4 (20–24). In this sheet of 64 × 64 neurons, each neuron is color coded to the index of the whisker to which it responds maximally. The nearby neurons respond to the same whisker forming barrels as can be seen in the figure. **(C)** The whiskers which are being considered for pathological study is shown in red circle (C1–C3 and D1–D3).

The input to LISSOM is the activity of a network that represents the whisker pad of the rat. The whisker pad on the snout of rats is modeled as an array of 24 whiskers (Figure 3A; Chen-Bee et al., 2012). Each blue dot represents one whisker and is identified using the row and column coordinates with numerals 0–4 representing columns and A-E representing the rows. Stimulation of a single whisker is modeled as a two-dimensional Gaussian function with peak at the location of the whisker being stimulated. The amplitude of the peak of the Gaussian function defines the amplitude of the whisker stimulation (Supplementary Figure 1). The equation for two-dimensional Gaussian function (Supplementary Equation 1) and the images of input stimulus at different amplitudes are given in Supplementary Material.

### Modeling the Neural Layer

The neural layer, which represents the V4 layer of rat whisker barrel cortex (Petersen, 2007), is modeled using a LISSOM, a biologically plausible model of cortical activity. The LISSOM is a two-dimensional network of neural units arranged on a grid as shown in Figure 2B. Each neural unit is connected to a small region of the input known as a receptive field using trainable afferent weights. Each neural unit receives an afferent signal which is a weighted sum of the intensity values in the receptive field. The neural units are also laterally interconnected using trainable lateral weights in such a way that the neighboring units within a specified radius (excitatory radius) of any given neural unit supply excitatory input and those units which lie outside the excitatory radius but within a specified outer radius (inhibitory radius) of provide inhibitory input to it. All the input signals are summed up and passed through a sigmoidal activation function. This center-surround pattern of lateral connections induces competition and helps in the formation of topographic maps as seen in the rat whisker barrel cortex.

The neural substrate for the LISSOM architecture is constrained so that the entire barrel cortex has a parabolic outline mimicking a real barrel cortex. The parabolic constraint is essential to produce barrels with an organization that is similar to the real barrel cortex. Interestingly, a similar curvilinear boundary constraint was found to be critical to model retinotopic map formation (Philips and Chakravarthy, 2017). The constrained LISSOM, when trained on the whisker stimulation input, forms topographic maps similar to the whisker barrel map seen experimentally (Petersen, 2007).

### Modeling the Vascular Layer

The vascular layer is modeled as a 3-dimensional tree structure with arteries branching first into penetrating arterioles and the arterioles branching into capillaries. The big pial artery gives rise to 16 smaller arteries and each of the arteries further branches into 16 arterioles giving rise to a layer of 256 penetrating arterioles. Each of the penetrating arterioles gives rise to 16 capillaries altogether forming a capillary bed with 4,096 capillaries. The capillary bed aligns itself with the neural layer. Like in the case of neural layer, each capillary is indexed by its location on the two-dimensional grid (Figure 2C). The assumption is that for every neural unit, there exists a capillary. It is at the level of capillaries that the oxygen exchange takes place. In this model, we assume that the capillaries, as well as arterioles, dilate in response to the neural activity. Each capillary is connected to a small receptive field (perfusion field) in the neural layer bidirectionally by an untrained weight matrix defined by two-dimensional Gaussian functions (Figure 2C). The Gaussian weight distribution ensures that the neurons influence the nearest capillaries and in turn get maximum nourishment from the nearest vessels. One assumption that we make here is that each vessel (capillary) is influenced by a neighborhood of neural units and the radius of this neighborhood (receptive field/perfusion field) is taken as approximately half of the perfusion field observed for penetrating arterioles (400 μmX400 μm) (O’Herron et al., 2016) since we do not have exact biological values identified for the perfusion field of capillaries. The standard deviation of the Gaussian weight distribution is fixed such that each capillary perfuses roughly 200 μm × 200 μm.

Figure 1B shows the complete schematic of the interaction between the neural and vascular layers. The neural activity modifies the compliance factor (β) which changes the pressure-volume relationship in the vessel. This causes a redistribution of the blood flow resulting in dilation of some vessels and constriction of others. The change in flow rate and the volume (V) influences the amount of oxygen saturation (S) in the blood. The amount of oxygen that diffuses out of the capillaries (OE) would be dependent on the gradient of partial pressure of oxygen (*PO*_2_) in the vessels and the neural tissues. This oxygen which reaches the tissues influences the production of Adenosine Triphosphate (ATP) by oxidative phosphorylation indicated by the cerebral metabolic rate of oxygen (CMRO_2_). The available Adenosine Triphosphate (represented by the variable ATP in this model) at the tissues in turn influences the threshold of neural firing.

The variables, β, volume, saturation, ATP, CMRO_2_, *PO*_2_, are described using a set of ordinary differential equations (ODEs). The method followed in order to integrate the set of ODEs with the neural network is explained in the Supplementary Material.

### Modeling the Neural Response

The LISSOM sheet consists of *nxn* neural units each of which is indexed using the row and column information in the grid. The net input of each neural unit is determined by a weighted contribution of afferent input, total excitatory input from the neural units in the excitatory radius, and the inhibitory input from the neural unit in the inhibitory radius. The equations and parameters used to generate input whisker stimulation using two-dimensional Gaussian function (Supplementary Equation 1), along with a few sample images of input stimuli (Supplementary Figure 1) are detailed in the Supplementary Material. The output of each neural unit is defined using Equations 1–3:

$$N_{i\,j}\left(t\right)=\sigma [P\left(\sum_{p\,q}W_{a\,f\,f_{i\,j,p\,q}}^{n\,I}I_{p\,q}\left(t\right)\right)$$

(1) $$\,+Q\left(EXC_{i\,j}\right)-R\left(INH_{i\,j}\right)-\varphi_{i\,j}]$$

(2) $$E\,X\,C_{i\,j}\,\left(t\right)=\sum_{k\,l}W_{i\,j,k\,l}^{e\,x\,c}\,N_{k\,l}\,\left(t-1\right)$$

(3) $$I\,N\,H_{i\,j}\,\left(t\right)=\sum_{k\,l}W_{i\,j,k\,l}^{i\,n\,h}\,N_{k\,l}\,\left(t-1\right)$$

where P, Q, and R are constants; $W_{a\,f\,f}^{n\,I}$ is the afferent weight stage from the input layer I to neural layer; *W*^*exc*^ is the excitatory weight stage; *W*^*inh*^ is the inhibitory weight stage; (*i*,*j*) denotes the index of a neural unit in the two-dimensional grid; (*k*,*l*) denotes the index of a neural unit in the neighborhood which gives excitatory or inhibitory projections to neural unit at location (i,j) depending on their proximity. σ is the activation function defined as,

(4) $$\sigma \,\left(s\right)=\left\{ \begin{array}{c} 0\;s\leq \theta_{\text{L}}\\ \frac{\left(s-\theta_{L}\right)}{\left(\theta_{U}-\theta_{L}\right)}\;\theta_{L}<s<\theta_{U}\\ 1\;s\geq \theta_{U} \end{array}\right.$$

where θ_*L*_*and*θ_*U*_ are the lower and upper thresholds of the sigmoid function. In Equation 1, φ_i,j_ is a threshold variable whose value depends on the available ATP in the neural tissue. In this model, the notation ATP represents the concentration of ATP. At rest, ATP is ATP_*ref.*_

(5) $$\varphi_{i,j}=\left\{ \begin{array}{c} \mu_{L}\;A\,T\,P_{i,j}\geq A\,T\,P_{t\,h\,r}\\ \mu_{H}\;A\,T\,P_{i,j}<A\,T\,P_{t\,h\,r} \end{array}\right.$$

where *ATP*_*thr*_is the minimum ATP required for the proper functioning of neurons. *ATP*_*thr*_ is defined in terms of percentage drop in resting state ATP (ATP_*ref*_). μ_*L*_ and μ_*H*_ are set in such a way that the μ_*H*_ > μ_*L*_.

All weights are trained using Hebbian learning as shown in Equation 6 for training lateral connections using asymmetric learning and Equation 7 for afferent weight training:

(6) $$W_{k\,l,p\,q}\,\left(t\right)=\frac{W_{k\,l,p\,q}\,\left(t-1\right)+\eta \,X_{p\,q}\,\left(t-1\right)\,Y_{k\,l}\,\left(t\right)}{\sum_{u\,v}W_{k\,l,u\,v}\,\left(t-1\right)+\eta \,X_{p\,q}\,\left(t-1\right)\,Y_{k\,l}\,\left(t\right)}$$

where *W*_*kl,pq*_is the lateral connection (excitatory or inhibitory) from neural unit (*p*,*q*) to neural unit (*k*,*l*), η is the learning rate,*X*_*pq*_(*t*−1) is the settled presynaptic neural unit activity at instance *t*−1 and *Y*_*kl*_(*t*) is the settled activity of the neural unit (*k*,*l*) at time t.

(7) $$W_{i\,j,p\,q}\,\left(t\right)=\frac{W_{i\,j,p\,q}\,\left(t-1\right)+\eta \,X_{p\,q}\,\left(t\right)\,Y_{i\,j}\,\left(t\right)}{\sum_{u\,v}W_{i\,j,u\,v}\,\left(t-1\right)+\eta \,X_{p\,q}\,\left(t\right)\,Y_{i\,j}\,\left(t\right)}$$

where *W*_*ij,pq*_ is the afferent connection weight from input pixel (*p*,*q*) to neural unit (*i*,*j*), η is the learning rate,*X*_*pq*_(*t*) is the input at instance *t*and *Y*_*ij*_(*t*) is the settled activity of the neural unit (*i*,*j*) at time t.

### Modeling the Vascular Response

Each blood vessel is considered to be a cylinder with diameter (d) and length (l) depending on the level of branching from the pial artery. The values of diameter and length are adopted from Boas et al. (2008). The other important variables that define the vessel characteristics are the Resistance (R), Volume (V), Pressure at the center of each vessel (*P_e*), Pressure at the node (*P_n*) from which branching of vessels takes place, the compliance factor of the vessel (β), and Saturation of oxygen in blood (S). A capillary at the grid location (i,j) is characterized by its length (*l*_*i,j*_), resistance (*R*_*i,j*_) and diameter (*d*_*i,j*_).

The resistance of each vessel can then be calculated using Poiseuille’s law,

(8) $$R_{i,j}=\frac{128\,\varepsilon \,l_{i,j}}{\pi \,d_{i,j}^{4}}$$

where ε is the fluid viscosity. The neurovascular coupling arises from the compliance factor β which is a function of neural activity. This factor results in the vasodilation of the proximal vessels. The various pathways that contribute to the vasodilation are considered by incorporating a time delay for the neural influence on the compliance factor.

(9) $$\tau_{1}\,\frac{d\,\beta_{i,j}\,\left(t\right)}{d\,t}=-\beta_{i,j}\,\left(t\right)+\beta_{0_{i,j}}\,\left(1-\frac{n\,e\,t_{i,j}\,\left(t-\tau \right)}{k_{1}}\right)$$

where *net* ∈ [0,1] is the weighted sum of neural activity (N) over an area in the LISSOM, β_0_ is β at resting state and *k_1* is a constant.

(10) $$n\,e\,t_{i,j}=\sum_{k,l}W_{i\,j,k\,l}^{v\,n}\,N_{k,l}$$

where $W_{i\,j,k\,l}^{v\,n}$ is the weight of the connection from the neural unit at a location (*k*,*l*) in the neural layer to the vessel at the location (*i*,*j*) in the vascular layer. The strength of the weight from a neural unit (*k*,*l*) to a vessel (*i*,*j*)drops exponentially with the square of its distance from the vessel. Hence the weight matrix projecting to a vessel from an area in the neural layer is defined by a Gaussian weight distribution centered at the coordinate of that vessel. The weight of connection from a neural unit (*k*,*l*), to a vessel (*i*,*j*) is defined by:

(11) $$W_{i\,j,k\,l}^{v\,n}=A_{w}\,e^{\left(-\frac{{\left(k-i\right)}^{2}}{\sigma_{w\,x}^{2}}-\frac{{\left(l-j\right)}^{2}}{\sigma_{w\,y}^{2}}\right)}$$

where *A_w* is the amplitude of the Gaussian, and σ_*wx*_ and σ_*wy*_ are the standard deviations. We allowed the dilation of a given penetrating arteriole to depend on an average of the effective neural activity felt by the branching capillaries originating from that arteriole.

The pressure at the center of the vessel (*P_e*) and volume (V) of the vessel are assumed to be related linearly as follows,

(12) $$P_{e_{i,j}}=\beta_{i,j}\,V_{i,j}$$

An increase in the neural activity causes a pressure drop in the proximal vessels enabled by a reduction in the compliance factor β. The pressure drop caused at the edge(s) due to the neural activity causes a redistribution of nodal pressures. The redistributed pressure is calculated by rewriting flow balance at a node in terms of nodal pressures and edge pressures.

The change in pressure, in turn, redistributes the flow of blood into the vessel and out of the vessel, building up the volume in some vessels. The flow of blood in and out of the vessels is calculated as follows (Boas et al., 2008).

(13) $$F_{i\,n_{i,j}}=\frac{P_{n\,1_{i,j}}-P_{e_{i,j}}}{R_{i,j}}$$

(14) $$F_{o\,u\,t_{i,j}}=\frac{P_{e_{i,j}}-P_{n\,2_{i,j}}}{R_{i,j}}$$

where *P*_*n1*_ is the nodal pressure at the mother node of a branch and *P*_*n2*_ is the nodal pressure at the child node of the branch. At rest, *F*_*in*_=*F*_*out*_. Neural activity induced changes in pressure disturbs this equality causing vasodilation or constriction. This causes a change in the volume (Boas et al., 2008) given by,

(15) $$\frac{d\,V_{i,j}}{d\,t}=F_{i\,n_{i,j}}-F_{o\,u\,t_{i,j}}$$

This change in volume and flow rate causes a change in oxygen saturation in the vessels. The rate of change of oxygen saturation depends on the difference in flow rate at the inlet and outlet at each vessel, the amount of oxygen extracted from the vessel (*OE*^*v*^), and the volume of the vessel. The saturation at the inlet (*S*_*in*_)of any vessel is assumed to be the saturation at the node from which it branches. The saturation at the outlet (*S*_*out*_) is again assumed to be the same as the saturation at the node to which it is connected at the bottom. The nodal saturations are calculated using the oxygen flow balance equation. At any node, the mass is conserved: the products of flow and saturation terms should add up to zero. The saturation of blood depends on the direction of the current flow. It is crucial to identify the immediate source point of flow to get a balanced mass-flow condition at each node.

(16) $$\sum_{i\,n}F_{i\,n}\,S_{i\,n}=\sum_{o\,u\,t}F_{o\,u\,t}\,S_{o\,u\,t}$$

The rate of change of saturation is given by the following relationship derived from Boas et al. (2008).

(17) Vi,j⁢d⁢Si,jd⁢t=Fi⁢ni,j⁢Si⁢ni,j-Fo⁢u⁢ti,j⁢So⁢u⁢ti,j-O⁢Ei,jvγ-Si,j⁢(Fi⁢ni,j-Fo⁢u⁢ti,j)

where γ is a constant representing the concentration of hemoglobin. Once the saturation of hemoglobin (S) and the volume (V) are estimated, the concentration of total hemoglobin (HbT), oxygenated hemoglobin (HbO) and deoxygenated hemoglobin (Hb) an be calculated as follows.

(18) $$H\,b\,T_{i,j}=2.3\,V_{i,j}$$

(19) $$H\,b\,O_{i,j}=2.3\,V_{i,j}\,S_{i,j}$$

(20) $$H\,b_{i,j}=2.3\,V_{i,j}\,\left(1-S_{i,j}\right)$$

The change in oxygen saturation in a vessel brings about a change in the partial pressure of oxygen (*PO_2*) in that vessel. The empirical relation (Kelman, 1966; Severinghaus, 1979; Boas et al., 2008) between the oxygen saturation and partial pressure of oxygen is given below:

(21) $$P\,O_{2_{i,j}}=\exp\left(3.85\,\left(\text{log}\,\left\{{\left(\frac{1}{S_{i,j}}-1\right)}^{-1}\right\}\right)\,3.32-\frac{1}{72\,S_{i,j}}-\frac{S_{i,j}^{6}}{6}\right)$$

At the same time, the neural activity consumes oxygen in the tissues in order to avail energy *via* oxidative phosphorylation. The change in the metabolic rate of oxygen (*CMRO*_2_) at the tissue is normally estimated as a function of flow rate, oxygen extraction fraction, and the hemoglobin concentration (Buxton and Frank, 1997). But from the discussions in Thompson et al. (2003) and Chong et al. (2015), neural activity appears to be a more direct correlate of CMRO_2_. Considering the fact that the total oxygen flux also has an equal significance, CMRO_2_ is influenced by neural signal and vascular signal equally. Thus, it appears to be the key variable in modulating the feedback from vessels. In order to independently incorporate the effect of feedback from the vessel to the tissues, based on the available oxygen at its neighborhood, we suggest the following equation for the metabolic rate.

$$\tau_{2}\,\frac{d\,C\,M\,R\,O_{2_{k,l}}}{d\,t}$$

(22) $$=\left(C\,M\,R\,O_{2\,r\,e\,f_{k,l}}\,A\,v_{O\,2_{k,l}}-C\,M\,R\,O_{2_{k,l}}\right)+O\,E_{k,l}^{n}\,N_{k,l}\,k_{2}$$

where *k_2* is a constant and *Av*_*O2*_ represents a fraction of available oxygen in the tissues, which is calculated as a fraction of *PO*_2*tiss*_ (partial pressure of oxygen) available at the tissues to the resting value of *PO*_2_ at the tissue(*PO*_2*tiss*_*max*_).

(23) $$A\,v_{O\,2}=\frac{P\,O_{2\,t\,i\,s\,s}}{P\,O_{2\,t\,i\,s\,s\,\_\,m\,a\,x}}$$

Equation 22 describes CMRO_2_ changes in a manner similar to the several existing models (Jones et al., 2001; Boas et al., 2003; Mathias et al., 2017b).

The increased oxygen metabolism results in a dip in the partial pressure of oxygen at the tissues. The rate of change of partial pressure of oxygen at the tissues depends on the total oxygen flux to the tissues (*OE*^*n*^) from the vessels and the rate of oxygen metabolism (CMRO_2_).

(24) d⁢P⁢O2⁢t⁢i⁢s⁢sk,ld⁢t=1α⁢VC⁢M⁢R⁢O2k,l⁢(O⁢Ek,ln-C⁢M⁢R⁢O2k,l)

where α*V*_*CMRO*2_ is a constant which represents the volume of tissue where the extracted oxygen is consumed. A reduction in the partial pressure of oxygen at the tissue leads to an increase in the extraction of oxygen from the vessels. The partial pressure of oxygen at the tissues as felt by each vessel is taken as a weighted sum of the partial pressure of oxygen at a small neural area around each capillary, corresponding to the receptive field of the capillary. The weight matrix used is the same Gaussian weight matrix used for calculating the effective neural activity at each vessel.

(25) $$P\,O_{2\,t\,i\,s\,s_{v\,e\,s\,s\,e\,l_{i,j}}}=\sum_{k,l}W_{i\,j,k\,l}^{v\,n}\,P\,O_{2\,t\,i\,s\,s_{k,l}}$$

where *PO*_2*tiss*_*vessel*_*i*,*j*__denotes the partial pressure of oxygen at the tissues as seen by the vessel at (*i*,*j*). The gradient between the partial pressure of oxygen at the capillaries and the tissues leads to diffusion of oxygen from the capillaries to the tissues and is defined by the following equation.

(26) $$O\,E_{i,j}^{v}=k_{3}\,\frac{P\,O_{2_{i,j}}-P\,O_{2\,t\,i\,s\,s\,\_\,v\,e\,s\,s\,e\,l_{i,j}}}{w_{k}}$$

where k_3_ is constant. The oxygen extracted from the vessels (*OE*_*v*_) diffuses to the tissues. The oxygen flux into each neural unit is calculated as the weighted sum of the oxygen extracted from an area of proximal capillaries which cater to the neurons. We assume here that most of the oxygen required by the neurons is being exchanged at the level of capillaries. The oxygen extracted by the neural unit at (k,l) is given by,

(27) $$O\,E_{k,l}^{n}=\sum_{i,j}W_{k\,l,i\,j}^{n\,v}\,O\,E_{i,j}^{v}$$

where $W_{k\,l}^{n\,v}$a Gaussian weight distribution centered on the neural unit *at*(*k*,*l*) similar to $W_{i\,j}^{v\,n}$ defined in Equation 11.

Adenosine triphosphate is required to fuel neural firing activity. Most of the ATP available at the neural tissues is a result of oxidative metabolism (Lin et al., 2010). The cerebral metabolic rate (CMRO_2_), therefore, could be taken as an indication of ATP generation and the neural activity could be taken as a measure of ATP consumption. The relation between generation and consumption of ATP is assumed to be as shown below. ATP_*ref*_ is the resting state level of ATP at the tissues, and the CMRO_2ref_ is the cerebral metabolic rate during the resting state.

$$\tau_{3}\,\frac{d\,A\,T\,P_{k,l}}{d\,t}$$

(28) =(A⁢T⁢Pr⁢e⁢f-A⁢T⁢Pk,l)⁢(C⁢M⁢R⁢O2k,l-C⁢M⁢R⁢O2⁢r⁢e⁢fk,lC⁢M⁢R⁢O2⁢r⁢e⁢fk,l)-k4⁢Nk,l

where *k_4* is a constant. The first term ensures that the ATP values return to their resting values when there is no production and consumption. The second term indicates the net increase in ATP by considering the production of ATP proportional to the percentage of increase in CMRO_2_. The third term indicates the consumption of ATP proportional to the neural activity. The values of the parameters are given in Table 1.

**TABLE 1:** Values of parameters and constants used in the model.

Constants Values	(*The parameters of training LISSOM network are given in Supplementary Material)
*k_1*	10 for capillaries 1 for arterioles
τ_1_	0.5 s for capillaries 1 s for arteries
*k_2*	0.7
τ_2_	2 s
*k*_*3_i,j*_	510^−8^l(i,j)d(i,j) mmmol/mmHg/s l – Length of the vessel d – Diameter of the vessel
*k_4*	0.5
τ_3_	4 s
*W_k*	2 μm
α*V*_*CMRO*2_	3.6050l*mol*/*mmHg*
γ	2.3 × 10-9 mol/μl
τ	500 ms
*A_w*	1
(σ_*wx*_,σ_*wy*)_	(2,2)
μ_*L*_	0
μ_*H*_	5
ATP_*ref*_	2.5
P	2
Q	14
R	8
θ_*H*_	10
θ_*L*_	$\frac{\theta_{H}}{6}$

The above equations are solved using DDE23 and Euler method in MATLAB to find the hemodynamic responses to neural activity.

### The Training Paradigm

The neurovascular network is used to validate two experimental paradigms. The first one is the validation of the network under normal conditions. For this, the neurovascular network is trained on the whisker stimulation and the corresponding vascular response is compared to the experimentally observed vascular response (Devor et al., 2005). The second part is the study of how vascular pathology influences the neural network plasticity. This part of the work is verified using the experimental studies done on neonatal rats. The LISSOM network is trained using the whisker stimulation input (Gaussian activation centered at each whisker location) for 50 epochs. The trained LISSOM is then connected to the vascular network forming three-layered structure with input as the first layer, LISSOM as the second layer and Vascular bed as the third layer as shown in Figure 2D. In order to check the hemodynamic response, the stimulus is given at time *t* = 0 and retained for 1 s. The duration of the input stimulus is fixed to be 1 s just as in the experimental study of Devor et al. (2005) and the hemodynamic response is observed for 4 s.

Simulating the network with all 24 whiskers is computationally expensive. Hence for modeling pathological conditions, we consider a smaller network where the input layer is a portion of whisker pad with just six whiskers. The neural layer with dimension 8×8 represents three barrels each of C and D row whiskers (Figure 3C). This is fed by a capillary bed of size 8×8. Once the pathology is introduced, the afferent connections from the input layer to the neural layer are retrained to observe the influence of the pathological condition in topographic map formation.

The retraining of the LISSOM network is carried out in the presence of vascular feedback under four conditions: (i) Control, (ii) Lesion, (iii) Hypoxia –Lesion, and (iv) Hypoxia-Ischemia. In the control condition, the network is retrained in the presence of healthy vascular feedback using the input stimuli from all the six whiskers. In the lesioned condition, one row of the whisker is assumed to be lesioned and hence retraining is carried out with just the stimuli from intact whiskers. Vascular feedback is healthy in this condition. In the model, we assume that the C whiskers are lesioned (the whiskers C1, C2 and C3 in Figure 3C). Hence, we train the network only by stimulating the D whiskers. The hypoxic condition is simulated by limiting the inlet oxygen saturation to 70% as compared to 94% in the healthy controls (Sicard and Duong, 2005). The study is also carried out under a range of oxygen saturation from 30% inlet oxygen saturation to 94% inlet oxygen saturation. The retraining of the LISSOM layer is carried out using input stimulations of D whiskers, under the assumption that C whiskers are lesioned. The final condition is that the arterial ligation is carried out to simulate ischemia along with hypoxia. We simulate it by reducing the diameter of the pial artery along with reducing the inlet oxygen saturation to 70%. The retraining of the LISSOM network is carried out using only D whisker stimulation input. The stimulation is given for 2 s preceded and succeeded by 1 s of rest period before giving the next stimulus. The training is run for four epochs for all four conditions. One epoch accounts the simulation of the network for the entire duration of *T* = 4 s. The training happens at every 0.1 s interval. Hence four epochs would account to 160 iterations. In order to plot the response map for all the four conditions, the response of each neural unit is observed before passing through the sigmoid function. The neural unit is assigned the label of that input to which it shows the maximal response.

The retraining of the LISSOM network is carried out in the presence of vascular feedback under four conditions: (i) Control, (ii) Lesion, (iii) Hypoxia –Lesion, and (iv) Hypoxia-Ischemia. In the control condition, the network is retrained in the presence of healthy vascular feedback using the input stimuli from all the six whiskers. In the lesioned condition, one row of the whisker is assumed to be lesioned and hence retraining is carried out with just the stimuli from intact whiskers. Vascular feedback is healthy in this condition. In the model, we assume that the C whiskers are lesioned (the whiskers C1, C2 and C3 in Figure 3C). Hence, we train the network only by stimulating the D whiskers. The hypoxic condition is simulated by limiting the inlet oxygen saturation to 70% as compared to 94% in the healthy controls (Sicard and Duong, 2005). The study is also carried out under a range of oxygen saturation from 30% inlet oxygen saturation to 94% inlet oxygen saturation. The retraining of the LISSOM layer is carried out using input stimulations of D whiskers, under the assumption that C whiskers are lesioned. The final condition is that the arterial ligation is carried out to simulate ischemia along with hypoxia. We simulate it by reducing the diameter of the pial artery along with reducing the inlet oxygen saturation to 70%. The retraining of the LISSOM network is carried out using only D whisker stimulation input. The stimulation is given for 2 s preceded and succeeded by 1 s of rest period before giving the next stimulus. The training is run for four epochs for all four conditions. One epoch accounts the simulation of the network for the entire duration of *T* = 4 s. The training happens at every 0.1 s interval. Hence four epochs would account to 160 iterations. In order to plot the response map for all the four conditions, the response of each neural unit is observed before passing through the sigmoid function. The neural unit is assigned the label of that input to which it shows the maximal response.

## Results

### Simulation of the Neural layer

The neural layer was trained using whisker stimulations of various amplitudes (Supplementary Figure 1). The trained LISSOM network resulted in a response map as shown in Figure 3B. Each colored blob represents the area in the rat barrel cortex that responds to one particular whisker. The colormap has values from 1 to 24 (representing all 24 whiskers shown in Figure 3A) which indicates the neurons that respond maximally when the corresponding whisker is stimulated. For example, the neural units coded with number 1 correspond to whisker located at row A and column 0. Hence when this whisker is stimulated, the neurons identified with number 1 respond maximally. Studies on the whisker barrel cortex show a parabolic arrangement of barrels (Chen-Bee et al., 2012). By imposing an area constraint on the LISSOM sheet, we were able to reproduce the parabolic whisker barrel map seen experimentally. The calibration of the neural network to represent the whisker barrel cortex is detailed in the Supplementary Material (section 2.a).

### Simulation of the Hemodynamic Response to Whisker Stimulation

**FIGURE 4:**
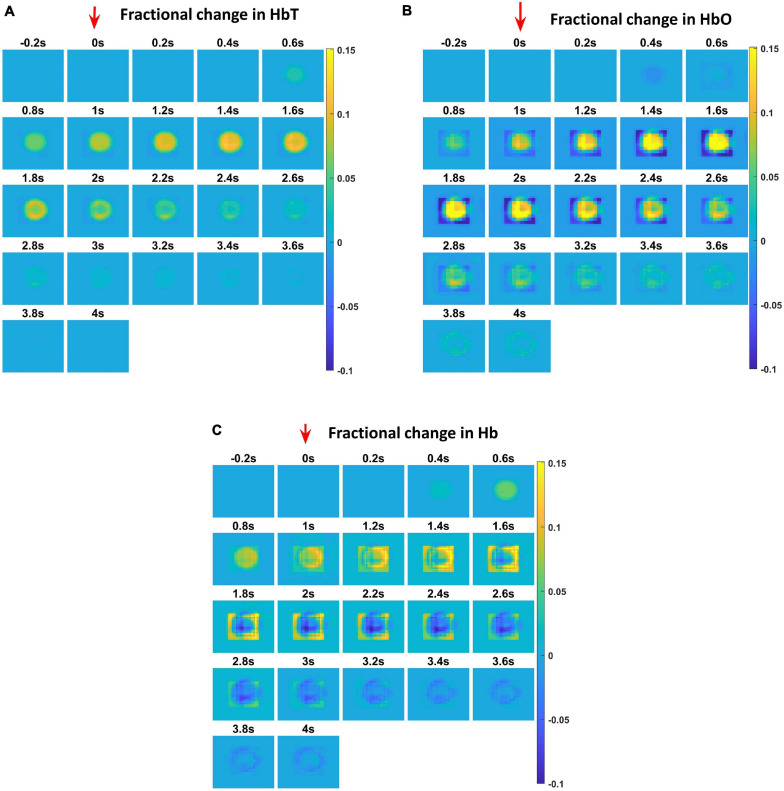
Each plot shows the time profile of different hemodynamic variables. The red arrow shows the time of presentation of stimulus. The color bar on the right shows the percentage change in value. **(A)** The time profile of HbT in the whisker barrel cortex—HbT is maximum at 1.6 s post stimulus presentation. **(B)** The time profile of HbO in the whisker barrel cortex. Soon after the stimulus, a dip in HbO can be observed at 0.6 s; HbO peaks around 1.6–2 s. **(C)** The time profile of Hb in the whisker barrel cortex. Soon after the stimulus, a slight peak in Hb can be observed at 0.6 s. Hb follows a continuous dip following that initial peak.

**FIGURE 5:**
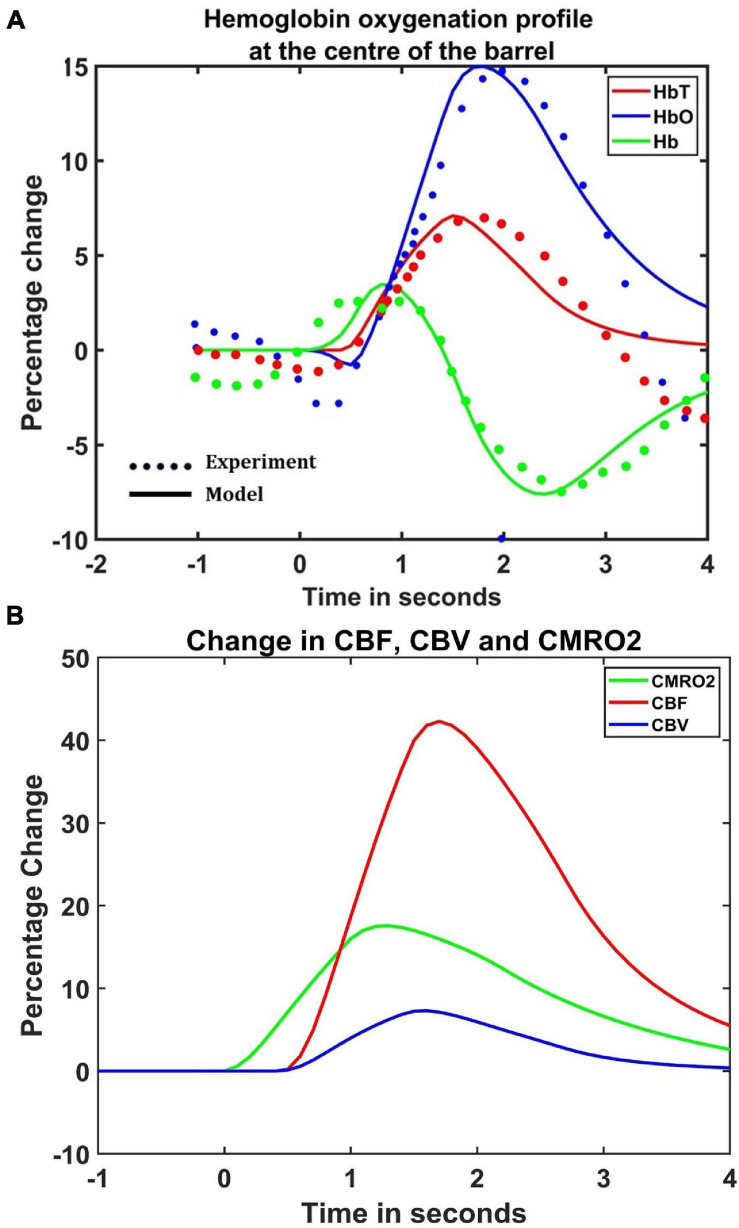
**(A)** Comparison of estimated hemodynamic response at the center of the activated barrel using the model with the experimental results. The dotted line plots the experimental values observed by Devor et al. (2005) and the solid line plots the values obtained from the model. **(B)** The percentage change in rCBF (red), rCBV (Blue), and CMRO2 (green) when the input of 1 s duration is presented at *t* = 0.

The hemodynamic response was observed after giving a stimulus of 1 s duration to the C3 whisker. It is characterized by the change in total hemoglobin (HbT) which is analogous to the change in volume, change in oxygenated hemoglobin (HbO) and change in deoxygenated hemoglobin (Hb). The variation of HbT with time is shown in Figure 4A. The red arrow on the figure indicates the time of presentation of stimulus. As expected, the capillary area corresponding to C3 whisker barrel area showed a response to the input stimulus by increase in HbT which also indicated an increase in volume or vasodilation. Vasodilation occurred after a delay of 0.5 s and this delay was optimized to match the experimental observation (Devor et al., 2005). The change in HbT reached a maximum at around 1.8 s post stimulus presentation (Buxton et al., 2004) and decayed slowly depending on the compliance factor. Since the duration of input stimulus is only 1 s, the compliance factor returned to the resting state according to Equation 9 once the input was removed. Our model was also able to capture the initial dip of HbO (Figure 4B) at around 0.6 s post-stimulus presentation as observed by Devor et al. (2005) and Berwick et al. (2008). HbO reached its peak at around 1.8–2 s post-stimulus presentation. Hb being complementary to HbO also showed an initial increase around 0.6 s after the presentation of the stimulus followed by a steady decrease (Figure 4C). HbO then slowly increased when HbT started to increase and Hb slowly decreased reflecting the hemodynamic response observed in rat whisker barrel cortex (Devor et al., 2005). Similar results were observed when input stimulus was given to any other whisker. Any whisker stimulation would result in a similar hemodynamic response pattern around an area in the capillary bed corresponding to the neural area in the whisker barrel cortex that responds to the whisker which is stimulated. The temporal variation of average hemodynamic response at the center of the principal barrel was compared with the experimental observation (Devor et al., 2005) as shown in Figure 5A. The variation of rCBF, rCBV, and CMRO2 in response to the input stimulation followed a pattern as shown in Figure 5B. The variation followed a similar trend as observed by earlier modeling studies (Aubert and Costalat, 2002).

The comparison of the model results with both spatial and temporal aspects of experimental observations is detailed in the Supplementary Material.

### Hemodynamic Response to a Whisker Stimulation at Various Locations in the Whisker Barrel Cortex

**FIGURE 6:**
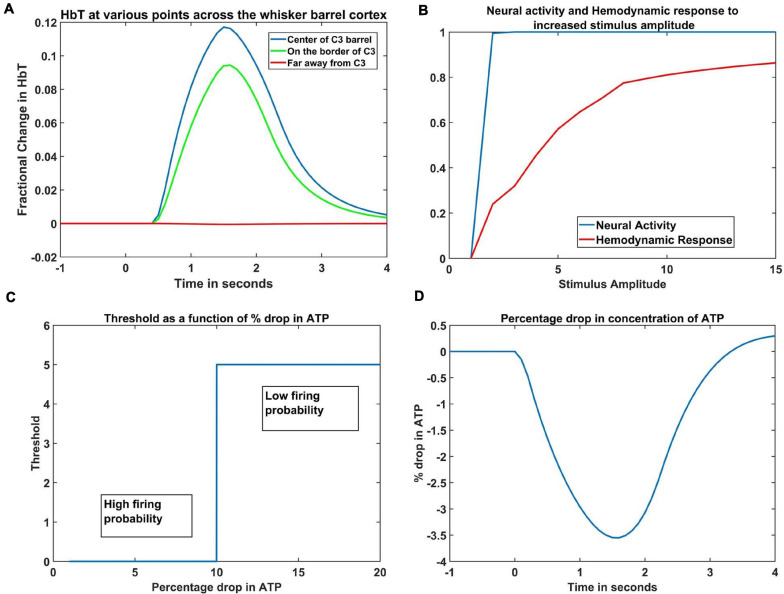
**(A)** Peak HbT observed 1.8 s post stimulus presentation to C3 whisker, (i) at the center of the barrel corresponding to the whisker (principal barrel) which was stimulated (blue), at the boundary of the principle barrel (green) and very far from the principal barrel (red). **(B)** The average of peak response of HbT (red) and the average neural activity (blue) around the principal barrel for different stimulus amplitudes. **(C)** Variation of threshold as a function of available ATP. **(D)** Observed variation in % drop of ATP around the principal barrel post stimulus presentation (stimulus is given at *t* = 0 s).

The hemodynamic response to a whisker stimulus varies throughout the capillary sheet depending on which whisker is stimulated. We considered the total area of the sheet as 4mm×4mm. The response of the entire sheet was observed after giving input stimulus at C3 whisker. The whisker barrel corresponding to the stimulated whisker is known as the principal barrel. Three points were identified in the capillary sheet, which is aligned to the whisker barrel cortex, one at the center of C3, another point at the boundary of C3 and D3, and one point at a long distance from center of C3, at the center of capillary area corresponding to A1 barrel. An area of dimensions 300 μm × 300 μm around the center of the principal barrel was considered as the region of interest (ROI). Figure 6A shows the average hemodynamic response (HbT) over approximately 300μm×300μm area surrounding each of the three points. The response to the stimulus occurred after a delay of 0.5 s. This accounts for the delay caused by the various vasodilatory pathways and is quantified here by the variable τ in Equation 9. Peaking of HbT response in the principal barrel occurred around 1.8–2 s after the presentation of input stimulus just as in the experimental study (Devor et al., 2005). A decrease in HbT outside the ROI was observed in the model, but it did not match the experimental results suggesting additional vasoconstriction in the nearby vessels.

### Hemodynamic Response Increases Beyond the Saturation of Neural Activity

With increasing input stimulus amplitude, the increase in neural response and hemodynamic response do not follow a similar pattern. Devor et al. (2005) observed that when the stimulus amplitude was increased continuously, the neural activity over an area saturated very soon, whereas the hemodynamic response continued to increase monotonically. We presented the network with input stimulations of various strengths defined by the amplitude of the Gaussian activity in the whisker pad. In Figure 6B, the X-axis denotes the amplitude of the input stimulus. The peak HbT was observed over an area surrounding the principal barrel, and the average of this peak HbT over that area was noted for each stimulus amplitude. The average neural activity was also noted around the same area for each stimulus amplitude. It was observed that the neural activity (blue) quickly reached saturation whereas HbT (red) increased slowly with stimulus amplitude. The model was able to capture the saturation of the neural activity and the monotonic increase of hemodynamic response over an area when presented with stimuli of increasing amplitudes.

### Change in ATP Variable Near the Neural Tissue in Response to Whisker Stimulation

The ATP variable in our model is not the biophysical ATP, but an abstract dimensionless variable that represents the concentration of the chemical. It is similar to the abstract ATP variable in Chhabria and Chakravarthy (2016). It is this variable that links the set of ODEs that define the vascular variables to the neural network variables. Since the variable ATP is an abstract term, we cannot compare the magnitude of its variation to the biological values, but we can see that its pattern of variation (Figure 6D) is quite similar with previous computational models (Aubert and Costalat, 2002; Ching et al., 2012) that plot the variation of ATP concentration. Our model was designed on an assumption that, under normal conditions, at the end of 1 s simulation, the ATP variable drops to around 3%- 4% of resting state value. This assumption was informed by the study of Aubert and Costalat (2002), where they compute the drop of ATP concentration for a sustained simulation that lasts around 360 s. To the best of our knowledge, there is no study that mentions the amount of drop in ATP following short whisker simulations, hence such an assumption is unavoidable.

The threshold of firing of a neuron depends on the available energy which is in turn determined by the concentration of ATP. In the current model, the threshold value was chosen depending on the percentage dip in ATP variable. If the ATP variable dropped more than 10% of its resting state value (ATP_*ref*_), the threshold was increased, lowering the firing probability of the neurons (Equation 1). The critical value was chosen as 10% of the resting state value of ATP variable, to fit the model to the studies of Ranasinghe et al. (2015). The dependency of threshold on ATP variable (Equation 5) is shown in Figure 6C. The idea of varying neural activation threshold as a function of ATP was consistent with prior modeling studies (Chhabria and Chakravarthy, 2016). Both the threshold and ATP are dimensionless variables, and they are the critical link between the vascular network and the neural network. The drop in the ATP variable starts soon after the input stimulus is given (at *t* = 0 s). The initial dip, followed by the slow rise is also in agreement with the study of Chhabria and Chakravarthy (2016).

### The Role of Vascular Pathology in Influencing the Plasticity of the Whisker Barrel Cortex

**FIGURE 7:**
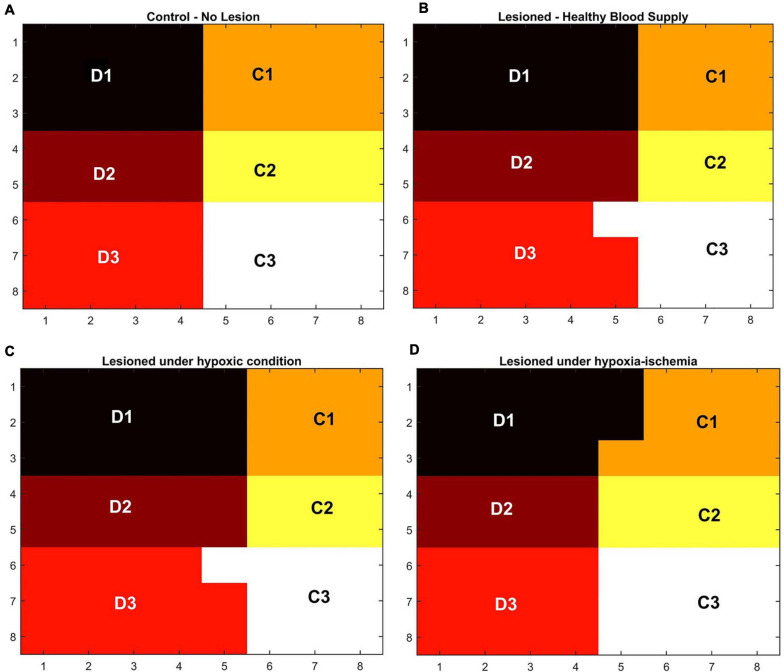
**(A–D)** Topographic map formation around a small area in the whisker barrel cortex under various conditions. **(A)** When the whiskers are intact, and blood supply is normal. **(B)** When the C1–C3 whiskers are lesioned, but blood supply is normal. **(C)** When the whiskers C1–C3 are lesioned and also the blood is hypoxic. **(D)** When the whiskers C1–C3 are lesioned and also under hypoxia-ischemia condition.

In order to observe the influence of vascular pathology on whisker barrel map reorganization, the LISSOM network was retrained in the presence of the vascular feedback. Since retraining the whole whisker barrel network was computationally intensive, a small area of whisker barrel cortex representing six whiskers, three whiskers each of C and D rows, was considered (indicated by the red circle in Figure 3C). The retraining in LISSOM is justified by considering the rats to be in the early stages of development where plasticity is high. During retraining, the map reorganizes if any metabolic mismatch causes a change in the neuronal response to the stimuli. The protocol used to study plasticity in the model was similar to the experimental protocol (Ranasinghe et al., 2015). The whisker barrel map reorganization was observed under four conditions: (1) Control, (2) Lesion, (3) Hypoxia –Lesion, and (4) Hypoxia-Ischemia, as explained in the “Materials and Methods” section. In Figure 7, a small area of whisker barrel cortex consisting of the neural units representing the C and D row whiskers are considered. In Figures 7A–D, each whisker barrel is identified by a color. The neural units which respond maximally when C1 whisker is stimulated identify the C1 barrel and are represented in orange color. Similarly, the neural units that form the C2 barrel are in yellow color, and those which form the C3 barrel are in white color. The neural units which identify the D1 barrel are represented in black color, those which form the D2 barrel are in dark maroon color, and the neural units which form the D3 barrel are in red color). The plasticity of the network in whisker lesion conditions was observed by the increase in the ratio of the total area, which represents D row (barrels D1, D2 and D3) to an area that represents C row (barrels C1, C2, and C3). This ratio is called theD/C ratio. Figures 7A–D shows the whisker barrel reorganization under conditions 1–4. It was observed that the areas of the C whiskers and D whiskers in the neural layer were almost equal when the retraining was done with healthy vascular feedback (Figure 7A), denoting equal representation for both the C and D whiskers. The lesioning of the C whisker led to the reduction in area representing C whiskers and increase in the area representing D whiskers (Figure 7B) displaying plasticity. Figure 7C shows that the plasticity was preserved under the hypoxic condition as observed by Ranasinghe et al. (2015). The fourth condition, which is the ischemia combined with hypoxia, caused a global reduction in blood flow, and due to inadequate feedback from the vessels, the neurons had a very low firing probability. This was due to the increase in the threshold value resulting from a low ATP value. Because of this low firing probability, plasticity did not occur. Hence, the response map remained unchanged as observed by Ranasinghe et al. (2015). The peak response to the stimulus was very low in the hypoxia-ischemia condition due to a large threshold value. Therefore, the response map shown in this case is not that of the output, but the net input obtained before passing through the sigmoid function. Basically, it is equivalent to displaying subthreshold activity of the neuron instead of the usual spiking response. This is similar to a situation where a neuron that is tuned to a particular whisker does receive the maximal input but due to the raised threshold, it is unable to fire. Because of this, the plasticity of the network was lost as observed in Figure 7D.

**FIGURE 8:**
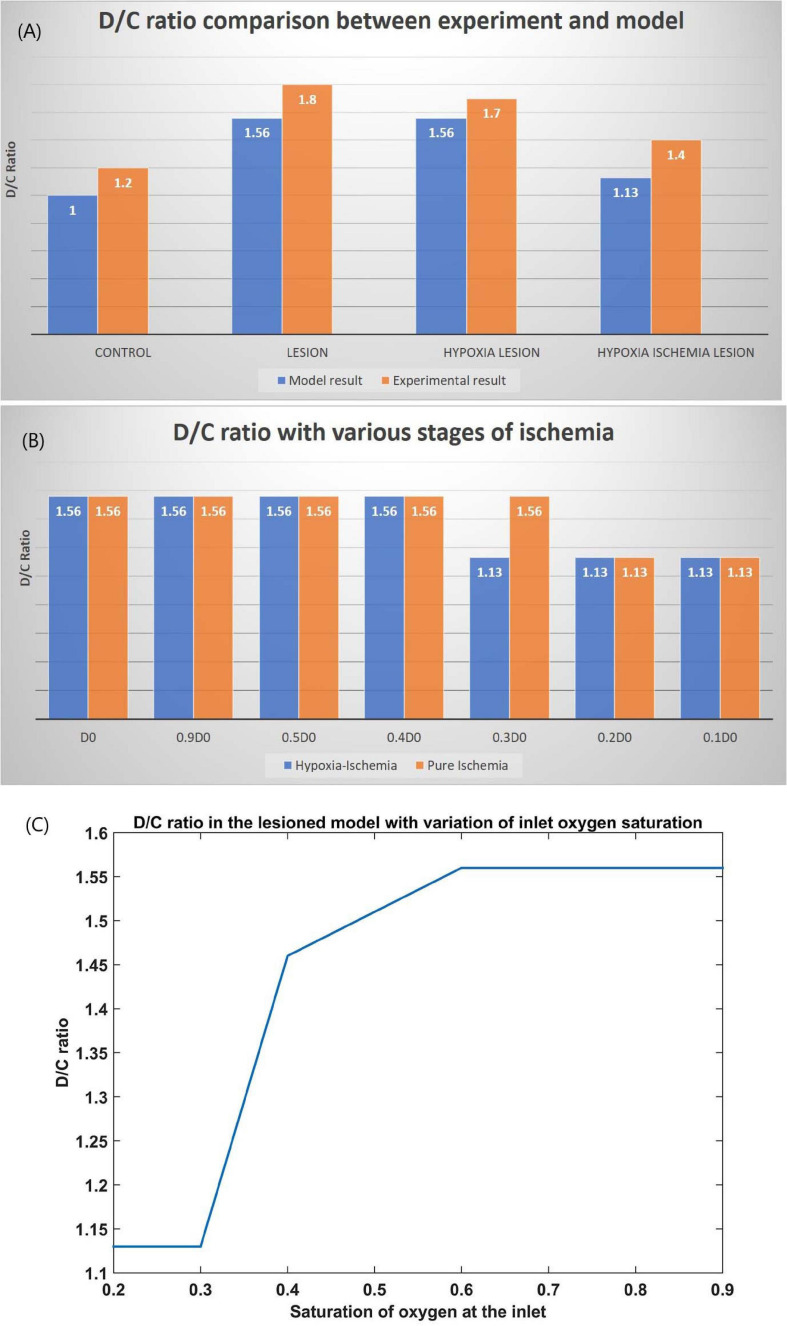
**(A)** Comparison of D/C ratio of experiment (Ranasinghe et al., 2015) and model under the four simulation paradigms. **(B)** The change in D/C ratio of a lesioned model at various stages of hypoxia-ischemia (blue) and purely ischemic condition (red). **(C)** The change in D/C ratio of a lesioned model at various percentages of oxygen saturation at the inlet.

The ratio of the cells representing the D whiskers to C whiskers under all four conditions was compared with that from the experimental observations in Figure 8A. The possible effect of stages of hypoxia-ischemia and pure ischemia on plasticity as observed by the model was shown in Figure 8B. The model supported the experimental observation that the hypoxic condition (oxygen saturation as low as 70%) alone does not affect the plasticity significantly. The model was also able to capture the absence of plasticity in Hypoxia-Ischemia condition. Thus, this experiment showed that short-term hypoxia does not compromise the neural functionality as compared to ischemia. The effect of various stages of hypoxia as predicted by the model is plotted in Figure 8C. As seen in Figure 8C, the model predicted an intermediate stage where plasticity is not completely lost, but as the inlet saturation drops below 60%, it impacts the plasticity. Similarly, there is a difference in how the plasticity is affected by a reduction in arteriolar diameter under normal oxygen supply and hypoxic condition (Figure 8B).

## Discussion

A biological neural network can carry on with its firing activity only if it has an adequate and timely energy supply. The vascular contribution to efficient neural performance has always been taken for granted. Our model attempts to capture the influence of retrograde signaling from vessels on the neural network characteristics. It assumes a middle ground between detailed biophysical models (Chander and Chakravarthy, 2012; Mathias et al., 2017a,b) and abstract models (Gandrakota et al., 2010; Philips et al., 2016). The key feature of the model is that it captures the feedback from the vascular layer to the neural layer, representing the dependence of neural activity on the energy substrates released by the cerebral vessels. The behavior of each neuron changes depending on the energy available to it and hence influences the network properties in a significant manner. Vascular structure, defined by a three-dimensional branching of blood vessels at different levels of hierarchy (arteries, arterioles, and capillaries), perfuses a two-dimensional neural sheet at the level of capillaries to retrieve the experimentally observed hemodynamic responses (Devor et al., 2003, 2005; Berwick et al., 2008) in the rat whisker barrel cortex. This model can be seen as a first step toward capturing the variations in hemodynamic responses observed in different areas of the brain (Lecrux and Hamel, 2016).

We chose LISSOM to model the barrels observed in the layer IV (L4) of the somatosensory cortex (S1) over the previous computational models of whisker barrel cortex like the works by Wilson et al. (2010) and Kremer et al. (2011), since our modeling requirement demanded a simpler version in order to reduce the computational complexity while incorporating the vascular dynamics during the training. The aim of the studies by Wilson et al. (2010) and Kremer et al. (2011) were to model the formation of direction selectivity map in the layer 2/3 (L2/3) barrel cortex, which is not included in our modeling objectives. Our intention was to understand the effect of vascular feedbacks on whisker barrel formation in normal and energy starved conditions. We hence formulated the whisker barrel cortex as simply as possible. In our model, the neural layer only required to map the principal barrels onto the whisker barrel cortex to simulate the barrels observed in the layer IV (L4) of the somatosensory cortex (S1) in rats. Hence the neural dynamics in the model was captured by the LISSOM model which is a simple yet biologically plausible model to simulate cortical neural activity (Miikkulainen et al., 2005). There is a more stable version of LISSOM, with gain control and adaptive threshold, GCAL (Bednar, 2012; Stevens et al., 2013) which makes the network robust to variation of contrasts in input stimulus. But we define the stimulus as a two-dimensional gaussian function centered around the whisker, with no variation in contrast. Hence even in the absence of gain control and adaptive threshold, the model would remain robust and stable over the inputs that are provided during the simulation.

Individual units of LISSOM encode the rate of neuronal firing over an area. The forward connections to the vessels carry the vasodilatory influence of the neural firing and are represented by a variable that affects the elasticity or compliance of the vessel wall. Oxygen consumption near the tissues is directly dependent upon the neural activity and available oxygen, which is a direct indicator of the metabolic demand. The feedback signal from the vessels to neurons is in the form of available oxygen for metabolism whose end result is ATP. The model treats the metabolic aspects of neurovascular interaction by considering first order dynamics for the cerebral metabolic rate of oxygen (CMRO_2_) with the synthesis term proportional to the net oxygen extracted from the vessels (OE) and the neural activity. ATP derived from this oxygen metabolism is again assumed to follow a first order dynamics with the synthesis term being governed by CMRO_2_ and the decay being proportional to the neural activity. (ATP) described in the Equation 28 presents a dimensionless quantity which replicates the characteristics of concentration of ATP. The profiles of CMRO2 and [ATP] are in agreement with the experimental observations (Hoge et al., 1999) and modeling studies (Aubert and Costalat, 2002). The dimensionless parameter representing [ATP] could be incorporated easily into the LISSOM network made up of sigmoidal neural units in the form of threshold of activation.

The proposed model which merges the biophysical metabolic quantities on the vascular side with abstract activation variables on the neural side was able to reproduce the hemodynamic response observed experimentally. The time profiles of the hemodynamic response-related variables (HbT, HbO, and Hb) were in agreement with the experimental observations (Aubert and Costalat, 2002; Devor et al., 2005). By introducing the delay of vasodilatory signal in reaching the vessels, the model was able to capture the immediate drop in oxygenated hemoglobin observed in the proximal vessels of excited neurons soon after the stimulus is given. The model was also able to replicate the change in hemodynamic variables with spatial accuracy. It was able to replicate the vascular dilation/HbT variation at various locations on the whisker barrel after stimulation of a single whisker. We can also control the amount of dilation in arterioles and capillaries individually to suit the literature (Biesecker et al., 2016).

As a first step to explore the influence of vascular feedback on neural network properties using this framework, we modeled the plasticity of the brain under conditions of hypoxia/ischemia in neonatal rats. The stimulation of a whisker normally activates a fixed area in the whisker barrel cortex. As long as all whiskers are intact, the area in the barrel cortex representing each of the whisker would be the same. But once a whisker is no longer active, the area representing that particular whisker shrinks and will be used to represent the other active whiskers. This plasticity, which is implemented in our model by Hebbian learning, is dependent on the vascular feedback. The retraining of the LISSOM layer with vascular feedback was carried out for four different paradigms, for four different vascular characteristics. Observing the response map of the retrained LISSOM network revealed the influence of vascular feedback on neural reorganization due to plasticity. When one row of whiskers is suppressed (C row), naturally, the plasticity of the neural network should allow the boundaries of neighboring (D row) whisker barrels to encroach into the area that previously represented the suppressed whiskers (C rows). But we observed that this encroachment by barrels of neighboring active whiskers, which is an indication of intact plasticity does not happen when the vascular feedback is not adequate. Ranasinghe et al. (2015) showed that neonatal rats had plasticity preserved as in controls, in hypoxic condition alone, but the plasticity was lost during hypoxia-ischemia condition. In the hypoxia-ischemia condition, the cortex was unable to respond to stimuli thereby preventing any plasticity. Our model was able to reproduce these effects on plasticity experimentally observed on neonatal rats. To the best of our knowledge, a study on how a step-by-step reduction of blood flow or a step-by-step reduction in oxygen concentration influences plasticity, and its consequences like cortical map formation, has not yet been carried out.

In the current model, we have assumed that more than 10% drop in ATP variable from the resting state value could reduce the firing probability of the neuron. Even though the network simulations using this choice agree with the available experimental studies, the model predicts a loss of plasticity at a hypoxic condition where inlet oxygen saturation is less than 60%. A careful experimental study that observes the effect of step-by-step variation of the inlet oxygen saturation level on plasticity will help confirm this assumption and the model can be used to predict the effect of various vascular phenomena on neural dynamics. This emphasizes the need for an extensive experimental investigation where the neural responses are observed under carefully calibrated vascular changes.

## Conclusion

As discussed by Hillman (2014), although several candidate mechanisms for neurovascular coupling have been identified, their integrated role has still not been understood. The vascular origin of many neurodegenerative diseases (Iadecola and Davisson, 2008; Iadecola, 2017; Kisler et al., 2017; Tarantini et al., 2017) points to the importance of understanding the proper functioning of neurovascular communication. Apart from this, the role of vessels in neural information processing is also a topic of debate (Moore and Cao, 2008; Pradhan and Chakravarthy, 2011). Vascular characteristics like vasomotion indicate that vessels have their own dynamics that may influence each other and the neuronal dynamics (Stergiopulos et al., 1998; Secomb and Pries, 2002; Pradhan and Chakravarthy, 2011). With an intact network layer structure for neurovascular interaction, by merely modifying the vessel model to incorporate active and intrinsic vascular dynamics,—and not just the vascular rhythms passively driven by neural inputs,—the effect of vascular dynamics on neural dynamics could be studied. Our model is an effort toward that direction.

The individual unit of the LISSOM network in this model is a sigmoid neural unit. This could be replaced by a network of spiking neurons that represent neural activity more accurately. The vascular network in this model has a tree structure of variable resistors in which the flow is analogous to the current flow. Also, neurovascular interaction could be mediated by modifiable connections, which makes possible multiple vascular adaptations like arteriogenesis (Buschmann and Schaper, 2000; Scholz et al., 2001) and angiogenesis (Krupinski et al., 1994; Beck and Plate, 2009) following ischemic conditions.

One drawback of this model is that we did not consider the lateral interaction among vessels, which, we believe, would be crucial for optimal distribution of blood flow. We also have not explicitly considered the role of astrocytes in the neurovascular coupling which might play an important part in both feedforward and feedback interactions between neurons and vessels, even though the generalized vasodilatory parameter takes care of that factor to an extent. The generalized vasodilatory parameter acts on the capillaries which are mediated by astrocytes and on the arterioles, which are controlled directly by neurons (Biesecker et al., 2016).

## Data Availability Statement

The original contributions presented in the study are included in the article/Supplementary Material, further inquiries can be directed to the corresponding author/s.

## Author Contributions

BK performed model designing, coding, analysis of the results, and manuscript preparation. AK performed model designing and coding. VC performed model designing, analysis of the results, and manuscript preparation. SP performed model designing and manuscript preparation.

## Conflict of Interest

The authors declare that the research was conducted in the absence of any commercial or financial relationships that could be construed as a potential conflict of interest.
