# Supplementary material for: A Network Architecture for Bidirectional Neurovascular Coupling in Rat Whisker Barrel Cortex
Source: Front Comput Neurosci. 2021 Jun 15;15:638700. doi: 10.3389/fncom.2021.638700 (PMC8241226; doi:10.3389/fncom.2021.638700)
Supplement: Supplementary file 1 [file Data_Sheet_1.pdf]

Supplementary material for A Network Architecture for Bidirectional Neurovascular Coupling in Rat Whisker Barrel Cortex

Authors:

Bhadra S. Kumar[1], Aditi Khot [2], V. Srinivasa Chakravarthy [1,*], S Pushpavanam[3],

Affiliations:

[1]Computational Neuroscience Lab, Dept. of Biotechnology, Bhupat and Jyoti Mehta School of Biosciences, IIT Madras, Chennai 600036, Tamil Nadu, India

[2] Dept. of Chemical Engineering, Purdue University, West Lafayette, USA

[3]Dept. of Chemical Engineering, Indian Institute of Technology Madras, Chennai 600036, Tamil Nadu, India

1. Laterally interconnected synergetically self-organizing map (LISSOM)

Laterally interconnected synergetically self-organizing map (LISSOM) is a network of laterally interconnected neurons which performs input driven self organization resulting in topographic map representation of input stimuli. The activity of each neuron depends upon the total weighted input it receives from the input layer and the lateral connections. It is calculated as shown in Eq.1 of the manuscript. During each input presentation, the activity of the neural layer takes several iterations before settling to a stable state. This is due to the lateral connections. The weight between two neurons are updated only when the sheet response settles down to a stable state. In eq. 6 and 7, X(t) and Y(t) represent the pre synaptic and post synaptic neuron connected by weight W. The values of X(t) and Y(t) should be used to update W only once the lissom sheet activity (eq.1) settles down. The number of iterations needed to settle down the LISSOM response is a model parameter.

The input to the LISSOM is a 2D Gaussian function as shown in fig. S1. The Gaussian function centered at one point represents the stimulation of the whisker at that point. The amplitude of the Gaussian represents the magnitude of the stimulation. The two dimensional Gaussian function at a point (x,y) is given by the following equation.

$$I(x,y) = A\, e^{-\left(\frac{(x-x_0)^2}{2\sigma_x^2}+\frac{(y-y_0)^2}{2\sigma_y^2}\right)} \quad \text{(S1)}$$

where A is the amplitude of the Gaussian, (x_o, y_o) is the location of the whisker being activated and σ_x and σ_y are standard deviations along x and y dimensions respectively.

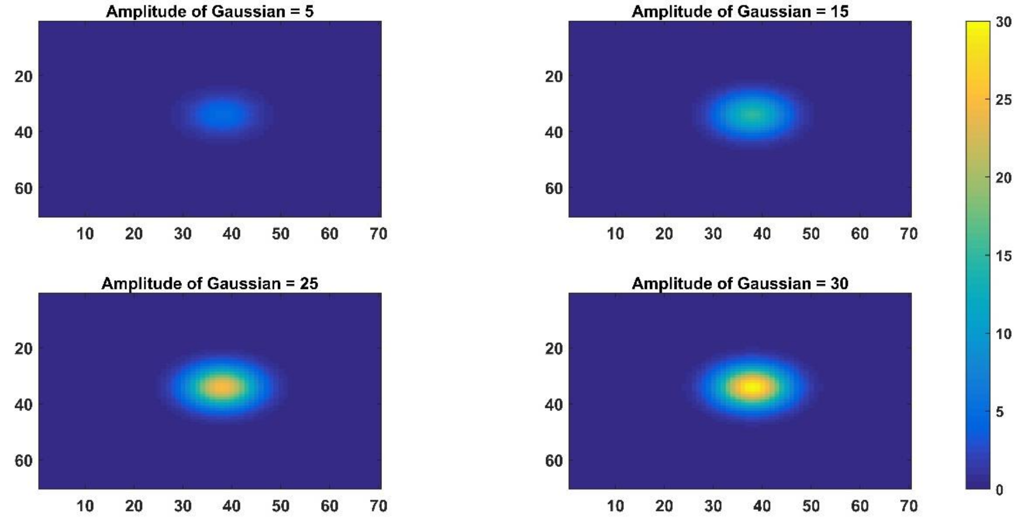

Fig.S1 : Gaussian input with different magnitudes

The output of a LISSOM unit before passing through the sigmoid function is given by

Z = P*Afferent projection + Q*Lateral excitatory projection+ R*Lateral Inhibitory Projection -threshold

The parameters of the LISSOM training are given below.

TABLE S1		
Parameter	Value of Big network	Value of Small network
LISSOM sheet size	64x64	8x8
Input Sheet Size	70x70	32x32
A	3 to 40	15
(σ_x , σ_y)	(4,4)	(2,2)
Receptive field	9x9	19x19

Stride	1	2
P	2	1
Q	14	10
R	8	8
Settling time	15 iterations	15 iterations
Epochs of training	50	50
Learning rate	0.01 for afferent and laterals	0.01 for afferent and laterals
Threshold	Resting ATP – 10%*Resting ATP	Resting ATP – 10%*Resting ATP

2. Comparison of model simulation with experimental results:

a. Spatial Comparison

The whisker barrels are mapped on to a 64x64 neural network as shown in fig.3B in the main manuscript. In the model, 64x64 pixels represent the 4mmx4mm area of whisker barrel cortex. Each pixel measures approximately 63μm. For calibration, we assume equal area of representation for each whisker. Thus, 4mmx4mm barrel cortex area has 24 whiskers which shows that one whisker is represented by an area of approximately 816μm X 816 μm, which is represented approximately by around 12x12 pixel area.

A sufficiently large amplitude of whisker activation can activate the neighbouring whiskers also as seen in(Berwick et al., 2008; Boas et al., 2008). Hence the spatial extent of barrel activation depends on the amplitude of input stimulus. In the model also this trend is observed as shown in figures S2.a to S2.d. As seen in image S2.d, for a sufficiently large amplitude of the input Gaussian curve, the spatial extent of increase in HbO spans over almost 30x30 pixels, which approximates to an area of 1.89 X 1.89mm in the whisker barrel cortex similar to the image 2.a of (Devor et al., 2005).

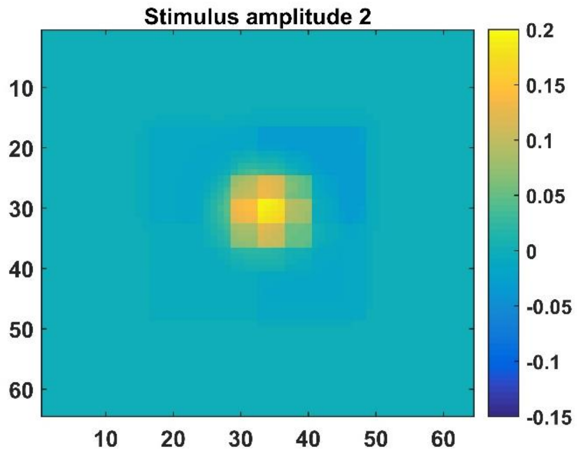

Fig. S2.a

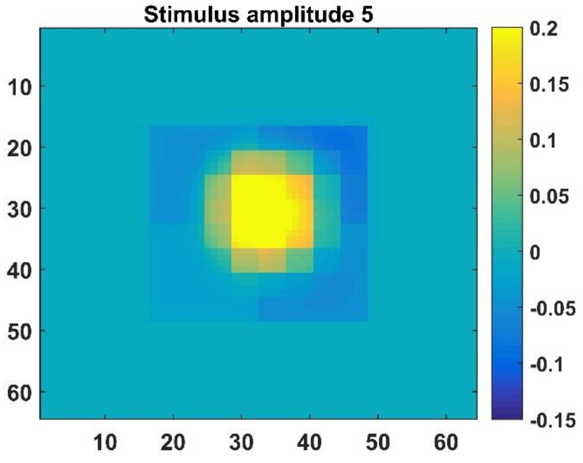

Fig. S2.b

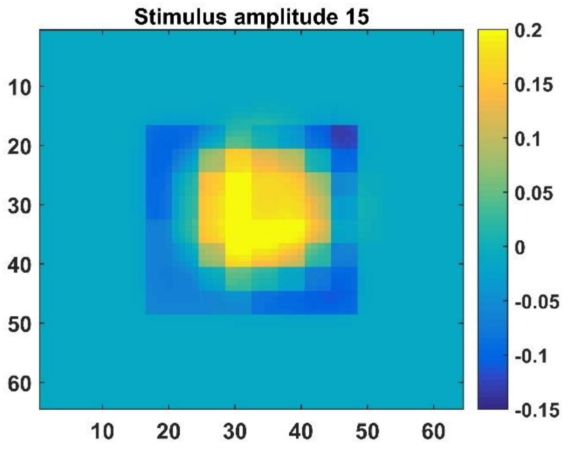

Fig S2.c

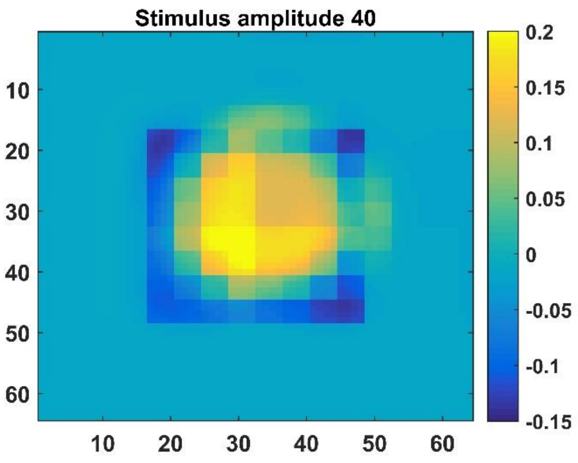

Fig.S2.d

Fig S2. HbO response across the whisker barrel cortex with a single whisker activation for different input stimulus

b. Temporal Comparison

The fig 5 of main document compares the experimental observation of hemodynamic variables at the centre of the active whisker when a stimulus of duration 1sec is given. The dotted lines are the experimental results (Devor et al., 2005; Boas et al., 2008)and the solid lines indicate the model output. The model result is the average change in hemodynamic variables in the principal barrel. The model is tuned such that the minimum input amplitude (Peak of Gaussian =3) gives an average hemodynamic response over the area of one barrel (12x12 pixels) similar to the experimental values plotted in fig.10 of (Boas et al., 2008).

3. Method by which the neural network is incorporated with the set of ODEs describing vascular dynamics

The dynamics of the variables, β, volume, saturation, ATP, CMRO2, PO_2, are described using a set of ODEs. The change in β is influenced by the neural output at each instant. But as far as the neural network is considered, there is no explicit time unit. The activity is defined by number of iterations. After each iteration, neural network updates the sheet response at depending on the input and available ATP which is again a result of the ODEs. It has to be ensured that the time scales of neural activity and vascular activity are properly aligned.

The ODE function is solved using Runge Kutta method, which has a dynamic step size depending on error tolerance. Since the neural output is called during each update of β, the number of times the LISSOM is updated depends on the step size and would lead to a mismatch in aligning the time scales of neural activity and vascular activity.

The ODE solver is defined such that even though it has a dynamic step size, it returns the value of the variables at a fixed interval of 0.1s. In order to make sure that the LISSOM also follows the similar time scales, LISSOM sheet is updated every 0.1s. If the step size of ODE is too small, the LISSOM will not get updated until after 0.1s from the previous instance of update and if the step size is larger than 0.1s, the LISSOM sheet is updated every 0.1s from the previous time instant of update before being used for updating β.

By this way, the vascular network defined by a set of biophysical equations is integrated with an abstract neural network model.

4. The change in HbT in the arterioles

The arterioles which are a level above the capillaries are also dilated in response to the neural activity. The change in radius can be read out from the plot of variation of total hemoglobin (HbT) (Eqn 18 in the main manuscript shows the relation between HbT and volume). Fig.S3 shows the variation in HbT in the arterioles during the rest, activity and post activity period similar to figure 4.a in main manuscript. The sheet size of individual subplots shown in fig.S3 is 16x16.

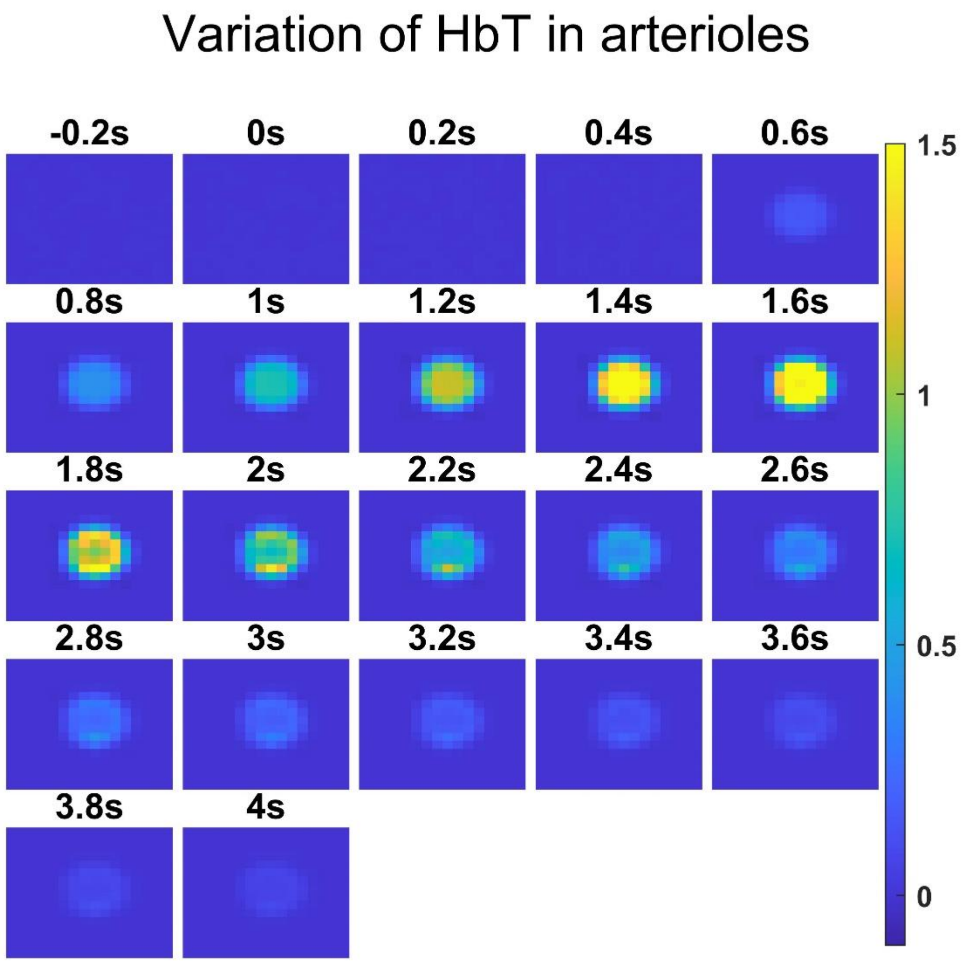

Fig S.3 The variation of total hemoglobin in the arterioles, one level above the capillaries. The sheet size is 16x16 The scale shows the fractional change in the concentration of total hemoglobin.

5. Variation of CMRO2 near the neural tissue as a response to the whisker simulation.

The variation in CMRO2 shown is for maximum stimulus amplitude of the input whisker stimulation. The input is given at time t=0 as shown by the red arrow. The stimulus duration is 1second.

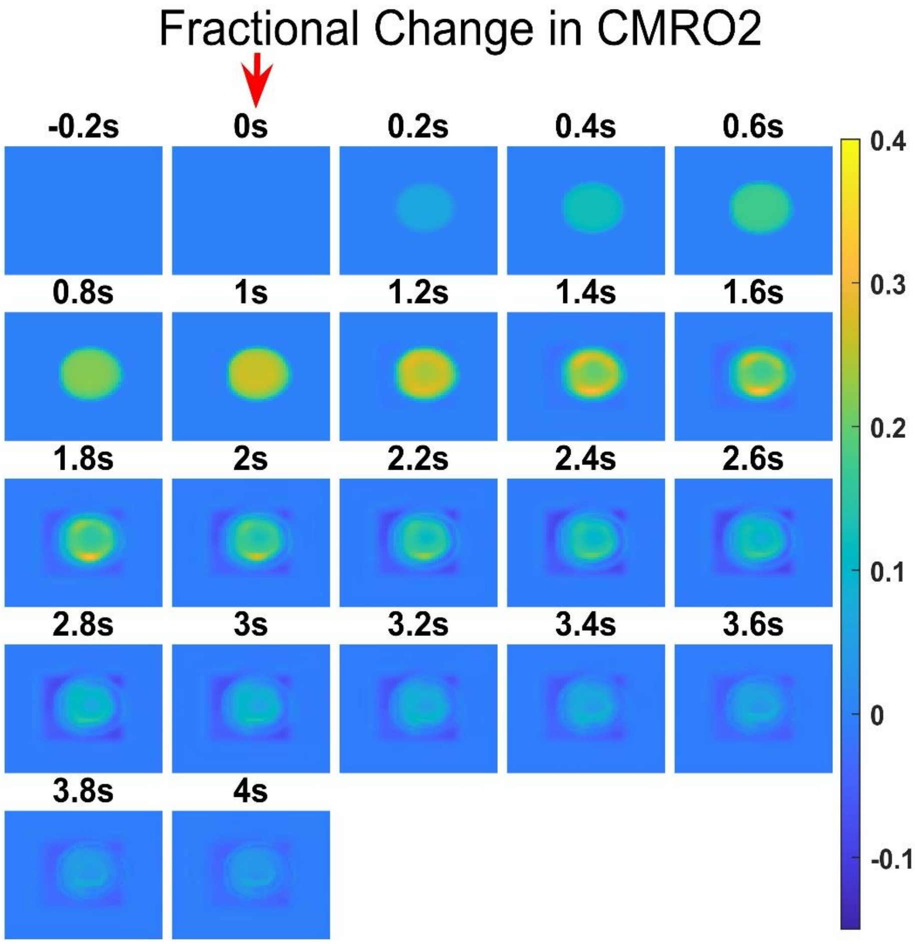

Fig. S4: The variation of CMRO2 at the neural tissues in response to whisker stimulation at t=0, shown in red arrow.

6. Variation of HbO in the capillaries during step by step reduction in inlet saturation.

The variation of HbO in capillaries with time according to the input presented is shown in fig. S5. The response of the vascular sheet (small network representing 6 barrels) to varied inputs are shown at different inlet saturations. The whisker is stimulated for 1second starting from time t=0.5 seconds and lasts till t=1.5 seconds. The retraining of the neural network in the presence of the vascular feedback is carried out while stimulating the active whiskers one at a time maintaining interstimulus interval of 1s. The higher activity seen in the t=0.1 in the images shown below is the residual HbO from the previous input presented to the network. As can be seen in images, the HbO change is very low at lower inlet saturations.

HbO at capillaries S0=0.94

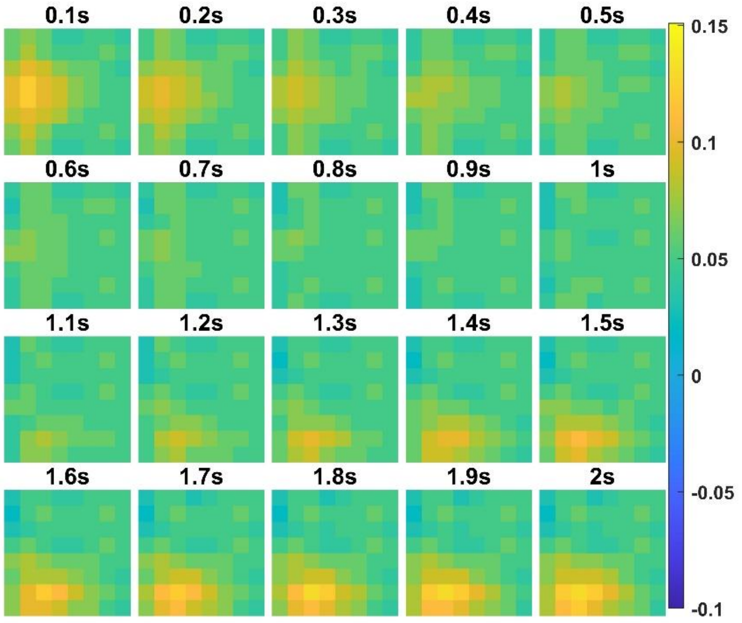

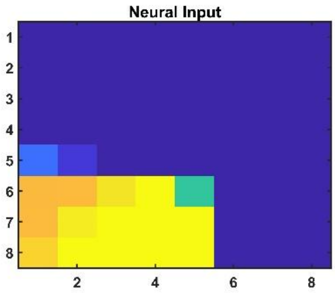

HbO at capillaries, S0=0.7

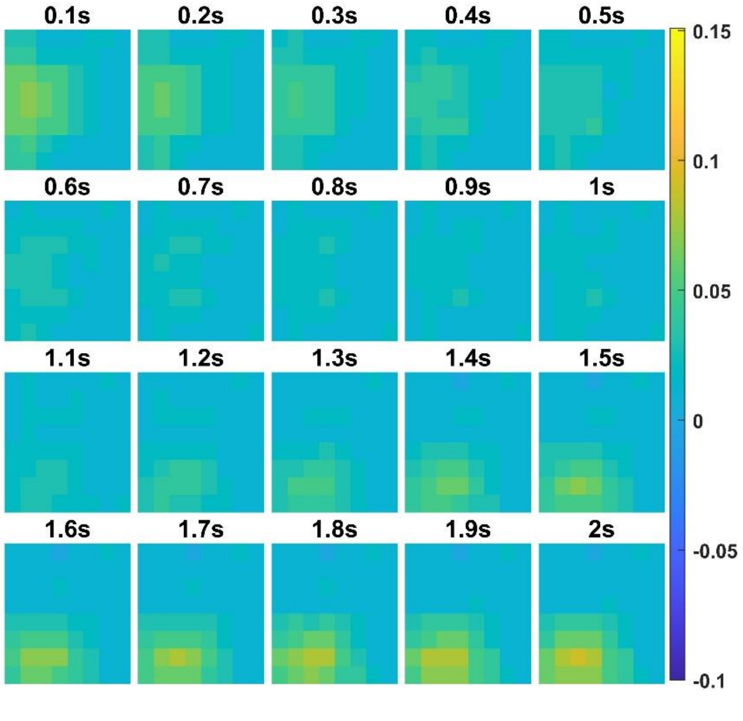

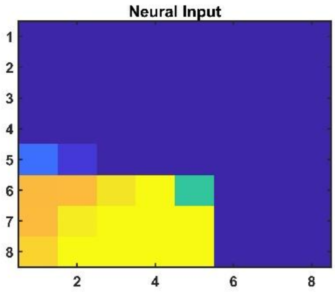

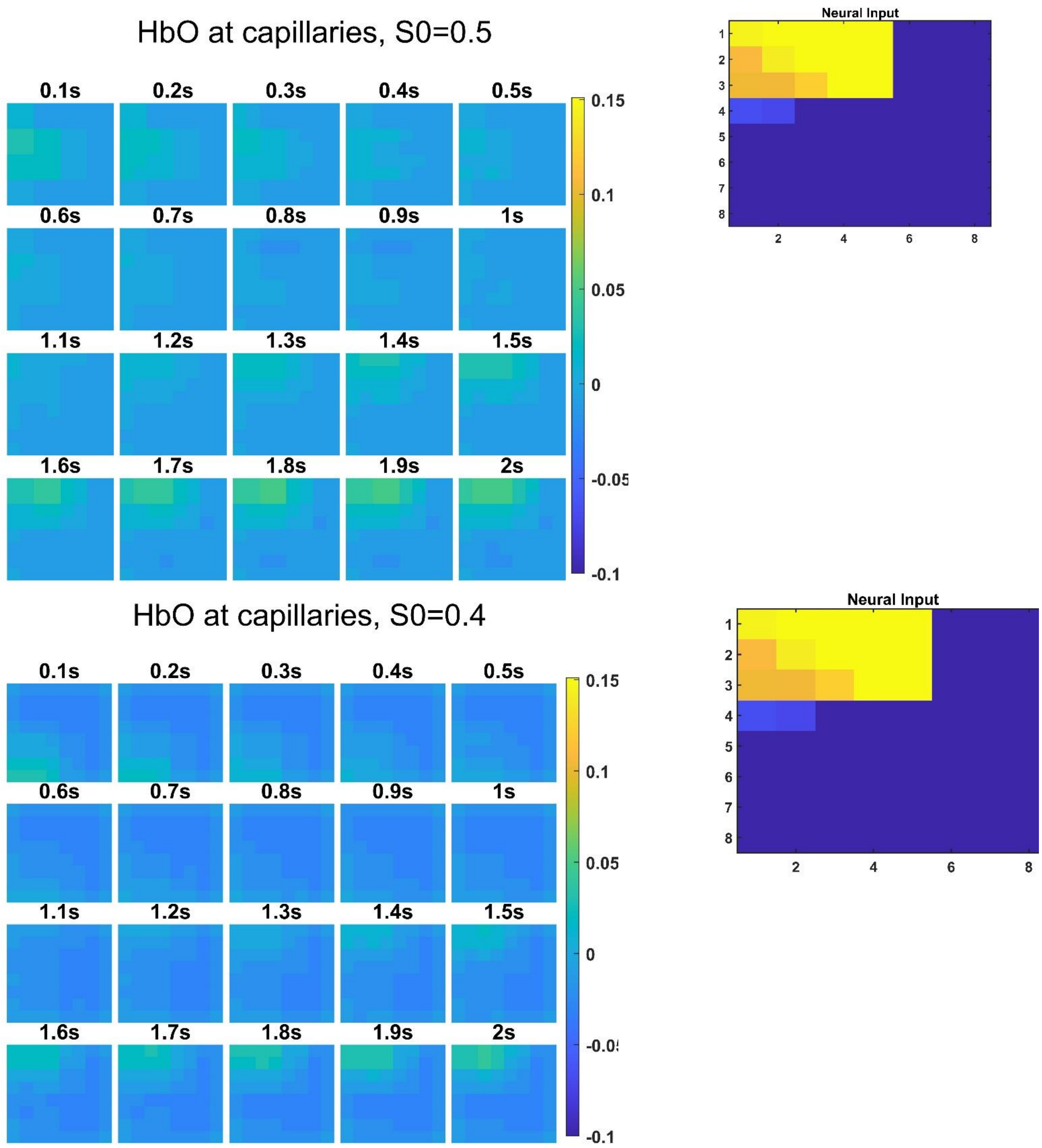

Fig.S5 Variation of HbO on presenting input stimulus at different inlet saturations
